# The relationship between workplace psychosocial environment and retirement intentions and actual retirement: a systematic review

**DOI:** 10.1007/s10433-018-0473-4

**Published:** 2018-04-19

**Authors:** Peter Browne, Ewan Carr, Maria Fleischmann, Baowen Xue, Stephen A. Stansfeld

**Affiliations:** 1 Centre for Psychiatry, Wolfson Institute of Preventive Medicine, Barts and the London School of Medicine and Dentistry, Queen Mary University of London, Charterhouse Square, London, EC1M 6 BQ UK; 2 Department of Epidemiology and Public Health, University College London, 1-19 Torrington Place, London, WC1E 7HB UK; 3 Department of Biostatistics and Health Informatics, Institute of Psychiatry, Psychology and Neuroscience, King’s College London, London, SE5 8AF UK

**Keywords:** Psychosocial work characteristics, Systematic review, Retirement behaviour, Retirement intentions

## Abstract

Psychosocial work characteristics are potential determinants of retirement intentions and actual retirement. A systematic review was conducted of the influence of psychosocial work characteristics on retirement intentions and actual retirement among the general population. This did not include people who were known to be ill or receiving disability pension. Relevant papers were identified by a search of PubMed, PsycINFO and Web of Science databases to December 2016. We included longitudinal and cross-sectional papers that assessed psychosocial work characteristics in relation to retirement intentions or actual retirement. Papers were filtered by title and abstract before data extraction was performed on full texts using a predetermined extraction sheet. Forty-six papers contained relevant evidence. High job satisfaction and high job control were associated with later retirement intentions and actual retirement. No consistent evidence was found for an association of job demands with retirement intentions or actual retirement. We conclude that to extend working lives policies should increase the job control available to older employees.

**Electronic supplementary material:**

The online version of this article (10.1007/s10433-018-0473-4) contains supplementary material, which is available to authorized users.

## Introduction

Across Europe, employment rates among older workers (55–64) increased from 38 to 53% between 2006 and 2016 (ONS 2016); however, early retirement before statutory pension age remains common. Most workers exit the labour market before reaching statutory pension age, which impacts detrimentally on the economy (PRIME 2014). With fewer people in work and more people claiming pensions, population ageing will place strain on welfare systems, and many governments are seeking to delay retirement. To promote extended working, it is therefore important to identify the determinants of retirement intentions and actual retirement. Decisions about when to retire have been linked to personal finances (de Wind et al. 2014), ill health (Virtanen et al. 2014; Ten Have et al. 2014) and psychosocial work characteristics (Wahrendorf et al. 2013; Carr et al. 2016). The latter are of particular interest because of their potentially modifiable nature (Karasek and Theorell 1990). Improving psychosocial work characteristics may also improve the well-being of employees.

To date, there has been no published systematic review of evidence regarding the influence of psychosocial work characteristics on retirement intentions and actual retirement. This paper seeks to review all available evidence and identify psychosocial work characteristics that are predictive of retirement intentions and actual retirement. We did not include physical working conditions as they are often not measured, or not well measured, in research studies on ageing, and we wished to concentrate the subject of our review on psychosocial work characteristics. We focus upon non-health exits from work, as opposed to disability pension. The impact of working conditions on people with existing illness may be different from the work-related factors in healthy retirement (Virtanen et al. 2014) and furthermore may differ by nature of ill health and type of occupation. Papers focussing on cohorts of individuals sharing a specific diagnosed illness are also excluded.

Several models have been proposed to explain the relationship between psychosocial workplace factors, employee health and retirement intentions and actual retirement. Karasek’s demand-control model (Karasek 1979) posits that the combination of high job demands (high work pace and conflicting demands) and low decision latitude (control over work, job variety and skill use) gives rise to job strain. According to this model, job demands create stress for the employee, but the effects of these demands are mitigated by the amount of freedom the employee is given in choosing how to meet them (‘decision latitude’). Johnson and Hall subsequently proposed the addition of social support to the demand-control model (Johnson and Hall 1988), whereby social support buffers the negative effects of high job demand. An alternative model proposed by Siegrist was the effort–reward imbalance (ERI) model (Siegrist 1996). This states that the combination of high effort (due to job demands and personal motivation) and low reward (e.g. poor salary, lack of opportunities for promotion) results in effort–reward imbalance that contributes to reduced employee well-being and ill health. More recently, Bakker and Demerouti outlined a job demands–resources (JD-R) model that considered all psychosocial variables to fall under two categories; either demands or resources (Bakker and Demerouti 2007). Job resources were defined as job attributes that stimulate personal growth, learning and development and contribute towards the achievement of work goals or reduce job demands. This model allowed any positive workplace factor to be considered a job resource, thereby permitting the study of a wider range of protective and deleterious workplace psychosocial factors.

Some additional psychosocial work characteristics have been studied which fall outside of the theoretical models mentioned above. These include job insecurity and organisational resources. Job insecurity measures people’s beliefs about how secure their job is into the future and how likely they are to lose their job (Ferrie et al. 2005). Organisational resources include positive treatment of employees by management including organisational justice, recognition of work by management and trust in management (Thorsen et al. 2016).

This review collates all relevant, published, quantitative studies and assesses the evidence for the association between psychosocial work characteristics and retirement intentions and actual retirement. To avoid presupposing that any one theoretical model is correct, we search for evidence relating to all major psychosocial work characteristics, and tables have been compiled for those where sufficient evidence is available. We include both cross-sectional and longitudinal studies of retirement and evaluate the association of psychosocial work characteristics with retirement intentions (i.e. before or beyond statutory pension age) and actual retirement (e.g. retirement before statutory pension age or remaining in work past statutory pension age). We hypothesise that high levels of job control, job satisfaction and social support are associated with later retirement intentions, reduced odds of exit from work or later age of actual retirement. High job demands, high job strain, high effort–reward imbalance and high job insecurity are hypothesised to predict earlier retirement intentions, increased odds of exit from work or earlier age of actual retirement.

## Methods

We carried out a systematic review of the association of psychosocial work characteristics with retirement intentions and actual retirement.

### Search strategy

We searched PubMed, PsycINFO and Web of Science for terms related to psychosocial work factors and retirement intentions and actual retirement (see ‘Appendix 1’ for full list of search terms). Terms were chosen based on the theoretical models discussed above. Filters for English language and scientific journal articles were applied to the searches. Searches were not restricted to any date range, so they spanned the full coverage of each database. All searches were conducted on 20/12/2016.

### Inclusion and exclusion criteria

Papers were deemed eligible if they were published in English, measured at least one psychosocial work factor and the outcome was either retirement intentions or actual retirement. Papers based on populations reporting specific illnesses or receipt of disability pension were excluded as different mechanisms may underpin retirement in the context of ill health. Papers were filtered for relevance in two stages: initially, based on title by a single reviewer (PB) and then according to their abstracts by two independent reviewers (PB and SS). Where the relevance of a paper was unclear based on title alone (in the first stage) that paper was passed to the second stage and relevance determined based on abstract. There was a high degree of concordance between reviewers. Papers over which there was disagreement were discussed at a project group meeting.

We extracted the following information from the included papers: study population, measurement of psychosocial work characteristics, retirement definition, sample size, gender balance, age range, duration of follow-up, adjustment variables and direction of evidence (with numerical results where available).

### Quality assessment

One reviewer (PB) carried out the initial quality assessment using the Newcastle–Ottawa Scale (Wells et al. 2009) which was compared with independent quality assessments of the same papers by the other two reviewers (SS and EC) and a consensus decision reached on quality scores. The Newcastle–Ottawa is a nine-point scale allocating points based on the selection of cohorts (e.g. representativeness of the sample; 0–4 points), the comparability of cohorts (e.g. whether the study controls for confounding factors; 0–2 points), the identification of the exposure (e.g. objectivity of exposure measurement) and the outcomes of study participants (e.g. independence of outcome measurement, adequacy of follow-up; 0–3 points).

### Analysis

Each reviewed paper presented one or more analyses involving psychosocial work characteristics and retirement outcomes. Analyses were reported as odds ratios, differences in means, hazard ratios, etc. All analyses addressing a given psychosocial work characteristic were gathered into a separate table, with results presented for retirement intentions and actual retirement in separate columns. We constructed tables for job resources, job demands, job satisfaction, social support, organisational resources, job insecurity and effort–reward imbalance. The tables for job resources, job demands and job satisfaction are presented in the main text because there was sufficient evidence to discuss subtypes of these characteristics. Tables for the other characteristics are presented in the supplementary material available online, and the evidence relating to these characteristics is discussed in the text. Our review combines evidence for men and women, because few papers stratified their analyses by gender.

### Operationalisation of psychosocial work characteristics

*Job resources* encompass several positive psychosocial work characteristics. In our review, they include job control, opportunities to develop, skill discretion, recognition at work, work variety and greater social cohesion. Job control is by far the most studied job resource, and consequently, it has been operationalised in many different ways. All of the following variables were categorised as measures of job control within this systematic review: decision latitude, decision authority, autonomy, predictability of work, influence at work and flexibility of work hours/place. Opportunities to develop was treated as a job resource, and the following were all categorised under this characteristic: availability of career development, availability of training, opportunities for growth, access to training, opportunity for role change and perceived schooling/training opportunities.

*Job demands* were operationalised by subjective stress/pressure, feeling overloaded, quantitative job demands, emotional job demands, work pace, role conflicts, time pressure and job strain. Job strain was defined as the ratio of job demands to job control (or in some cases, the difference between job demands and job control). There was only one analysis of job strain, and it was included under job demands in this review.

*Job satisfaction* included professional satisfaction, career satisfaction, work enjoyment, challenge at work, meaningfulness of work, reward in work, work providing active interest and job content plateau. Job content plateau is a negative characteristic of work defined as ‘the point at which a job becomes routine and boring, with the likelihood of not receiving further assignments of increased responsibility’ (Hofstetter and Cohen 2014). In this review, we treated it as an example of job dissatisfaction.

*Social support* was defined differently by almost every study which included it. This heterogeneity made the summary table less informative for this work characteristic, so that table has been moved to the online supplementary materials. The following were all categorised as measures of social support in this review: support from co-workers, support from supervisors, quality of leadership, team-working and perceived support from supervisors for working till age 65. The following were taken to be negative workplace characteristics indicative of low social support: exposure to bullying, conflicts in work and perceived pressure from colleagues to retire early. All studies which analysed social support without drawing more precise distinctions between different types of support were grouped under the heading ‘greater social support’.

*Organisational resources* included operationalisations of organisational support, organisational justice, management quality and organisational stimulation. Organisational injustice was treated as an operationalisation of a lack of organisational justice and therefore as an absence of an organisational resource.

There were some characteristics that did not feature in enough analyses to permit conclusions to be drawn about them. These characteristics feature in the summary tables, but they are not presented in their own tables in the main text (see supplementary online material for tables of these characteristics). *Effort*–*reward imbalance* was operationalised, in all studies, as the imbalance between effort expended at work (including commitment to work) and rewards received (in terms of being valued at work, salary, promotion prospects and job security). The psychosocial work characteristic *job insecurity* was measured by participants’ concerns about unemployment (e.g. ‘Are you worried about becoming unemployed?’) and the perceived possibility of demotion.

### Operationalisation of retirement outcomes

The associations between psychosocial work characteristics and retirement intention and actual retirement are presented based on the direction of evidence. We operationalised three possible outcome categories: (1) ‘Earlier retirement’ indicated that a given psychosocial work characteristic was associated with intended or actual early retirement (before statutory pension age), intentions to stop working or increased risk of labour market withdrawal. (2) ‘Null’ indicated a lack of statistically significant associations (at the 5% level) between a given psychosocial factor and retirement outcomes. (3) ‘Later retirement’ indicated that a given psychosocial work characteristic was associated with intended or actual retirement beyond statutory pension age or reduced risk of labour market withdrawal.

## Results

**Fig. 1:**
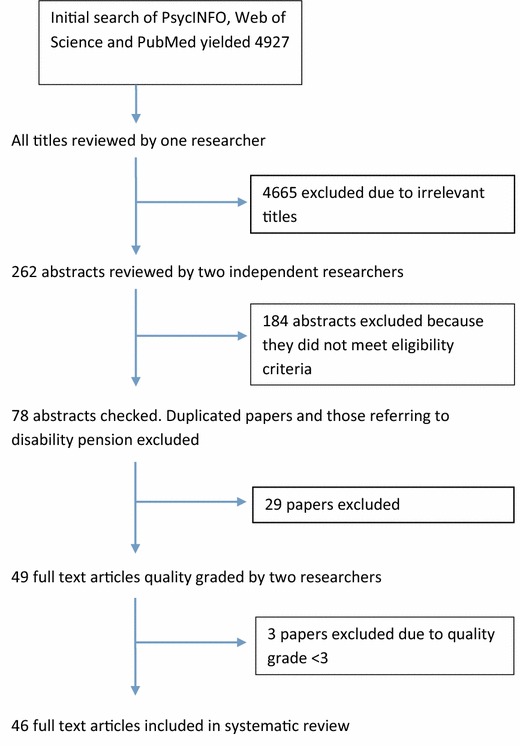
Flow diagram of studies selected for the systematic review

The initial search retrieved 4927 results, 4665 of which were excluded because their titles indicated they did not meet the eligibility criteria (Fig. 1). Two researchers independently reviewed the remaining 262 abstracts and 184 were excluded. At this stage, we also excluded studies of disability retirement. Of the remaining 78 papers, 49 were deemed relevant by two researchers on reading the full abstracts. These papers underwent independent quality grading by two researchers, and 3 were discarded due low quality (scoring below 3 on the Newcastle–Ottawa Scale). The final review included evidence from 46 papers. This included 22 cross-sectional and 21 longitudinal studies and three studies that were both cross-sectional and longitudinal. A comprehensive table containing all extracted data is available as an online supplement.

Table 1 presents the number of papers examining each psychosocial work characteristic and the number of analyses that were carried out, for both retirement intentions (92 analyses) and actual retirement (81 analyses). The number of analyses exceeds the number of papers because some papers contained analyses of multiple psychosocial work characteristics. Table 1 also summarises the direction of evidence, giving the number of the analyses supporting an association between each psychosocial work characteristic and ‘earlier retirement’ and ‘later retirement’ as detailed above.

**Table 1:** Summary of evidence for association of psychosocial work characteristics with retirement timing

Psychosocial factor (number of papers that include this factor)	Analyses of retirement intentions				Analyses of actual retirement			
	Direction of evidence			Total analyses of retirement intentions	Direction of evidence			Total analyses of actual retirement
	Earlier retirement	Null	Later retirement		Earlier retirement	Null	Later retirement	
Resources (38)	2	12	23	37	1	11	16	28
Demands (30)	8	11	–	19	2	18	2	22
Satisfaction (19)	2	2	12	16	–	5	5	10
Social support (17)	1	5	6	12	1	7	6	14
Organisational resources (6)	–	1	3	4	–	1	1	2
Effort–reward imbalance (5)	–	–	2	2	–	2	1	3
Job insecurity (3)	1	1	–	2	1	1	–	2
Total	14	32	46	92	5	45	31	81

Overall, we found good evidence for the association of positive psychosocial work characteristics (e.g. job resources, satisfaction, social support) with retirement intentions and actual retirement, but there was less evidence for the role of job demands. For other work characteristics (organisational resources, effort–reward imbalance and job insecurity), there was insufficient evidence to draw conclusions. For job resources, most analyses found greater resources to be associated with later retirement (23/37 analyses of retirement intentions, 16/28 analyses of actual retirement). For job demands, although some analyses (8/19) found high demands to be associated with intentions for earlier retirement, most analyses of actual retirement found no statistically significant association (18/22). Work-based social support was associated with later retirement intentions and actual retirement in 6/12 and 6/14 analyses, respectively. Results for subtypes of each work characteristic are presented in Tables 2, 3 and 4.

**Table 2:** Direction of evidence for analyses of job resources in relation to retirement timing

Type of job resource (number of papers that include this measure)	Analyses of retirement intentions				Analyses of actual retirement			
	Direction of evidence			Total	Direction of evidence			Total
	Earlier retirement	Null	Later retirement		Earlier retirement	Null	Later retirement	
Greater job control (33)^a^	–	9	13	22	–	8	10	18
Higher skill discretion (3)	–	1	–	1	–	1	1	2
Greater opportunities to develop (7)	1	–	5	6	1	1	3	5
More job resources (6)^b^	–	1	3	4	–	1	1	2
Recognition (2)	–	1	1	2	–	–	1	1
Work variety (1)	–	–	1	1	–	–	–	0
Greater social cohesion (1)	1	–	–	1	–	–	–	0

^a^Job control included ‘autonomy’, ‘influence at work’, ‘flexibility of working hours/place’, ‘predictability in work’, ‘decision latitude’ and ‘decision authority’

^b^This includes all studies which analysed job resources without drawing more precise distinctions between different types of resources. Where studies defined job resources in more detail, they appear on other rows of the table

### Job resources

Job control was assessed in 33 papers, with 22 analyses of retirement intentions and 18 analyses of actual retirement (Table 2). Greater job control was associated with later retirement intentions in 13/22 analyses (Virtanen et al. 2014; Wahrendorf et al. 2013; Ten Have et al. 2014; Carr et al. 2016; Sutinen et al. 2005; van den Berg 2011; Siegrist et al. 2007; Harkonmaki et al. 2006; Heponiemi et al. 2008; Suadicani et al. 2013; Stynen et al. 2016; Frins et al. 2016; Thorsen et al. 2012; Elovainio et al. 2005) and with later actual retirement in 10/18 analyses (Virtanen et al. 2014; Carr et al. 2016; Thorsen et al. 2016; Blekesaune and Solem, 2005; Roebroek et al. 2013; Clausen et al. 2014; Friis et al. 2007; Hintsa et al. 2015; Roebroek et al. 2015). Greater opportunities to develop were associated with later retirement intentions and actual retirement in 5/6 (Stynen et al. 2016; Thorsen et al. 2012; Henkens and Leenders 2010) and 3/5 (Thorsen et al. 2016; Herrbach et al. 2009; van Solinge and Henkens 2014) analyses, respectively. Other subtypes of resources were not analysed enough times for us to draw conclusions.

#### Job demands

Subtypes of job demand included subjective stress/pressure, feeling overloaded, quantitative job demands, emotional job demands, work pace, role conflicts, time pressure and job strain (Table 3). There were 23 analyses of job demands (which included role conflict and quantitative demands). Of these, 5/13 (Carr et al. 2016; Harkonmaki et al. 2006; Frins et al. 2016; Elovainio et al. 2005; Schreurs et al. 2011) found an association with earlier retirement intentions; 1/9 (Jensen et al. 2012) found an association with earlier actual retirement; and a further 1/9 found an association with later retirement intentions (Suadicani et al. 2013). Nine papers considered stress/pressure, with five analyses of retirement intentions and seven analyses of actual retirement. Most of these found no statistically significant association with either earlier or later retirement. There were 2/5 (Sutinen et al. 2005; Kilty and Behling 1985) and 5/6 (Thorsen et al. 2016; Blekesaune and Solem 2005; Robroek et al. 2013; Friis et al. 2007; van Solinge and Henkens 2014) analyses that found no association between stress/pressure and retirement intentions and actual retirement, respectively (whereas there were 3/5 (Henkens and Leenders 2010; van Solinge and Henkens 2014; Burnay 2008) and 1/6 (Friis et al. 2007) analyses supporting an association between stress/pressure and early intentions and actual retirement, respectively). Six papers included emotional demands with most analyses (5/6) (Stynen et al. 2016; Thorsen et al. 2016; Clausen et al. 2014; Oude Hengel et al. 2012; Lund and Villadsen 2005) producing null outcomes. One paper analysed the association of job strain with actual retirement and produced a null outcome (Virtanen et al. 2014).

**Table 3:** Direction of evidence for analyses of job demands in relation to retirement timing

Type of job demand (number of papers that include this measure)	Analyses of retirement intentions				Analyses of actual retirement			
	Direction of evidence			Total analyses of retirement intentions	Direction of evidence			Total analyses of actual retirement
	Earlier retirement	Null	Later retirement		Earlier retirement	Null	Later retirement	
Job demands (21)^a^	5	8	–	13	1	8	1	10
Stress/pressure (9)^b^	3	2	–	5	1	5	1	7
Emotional demands (6)	1	1	–	2	–	4	–	4
Job strain (1)	–	–	–	0	–	1	–	1
Total	9	11	0	20	2	18	2	22

^a^This includes all studies which analysed job demands without drawing more precise distinctions between different types of demands. It also includes the variables ‘role conflicts’ and ‘quantitative demands’ as these were felt to be operationalisations of job demands

^b^Stress/pressure includes ‘feeling overloaded’, ‘workload’, ‘work is too consuming’, ‘time pressure’, ‘pressure at work’, ‘busyness at work’ and ‘work pace’

#### Job satisfaction

There were 11 papers that considered a general measure of ‘job satisfaction’ (Table 4). In about half the studies greater, job satisfaction was found to be associated with later retirement intentions (4/8) (Schreurs et al. 2011; Burnay 2008; Pit and Hansen 2014; Oakman and Wells 2016), and in all the papers on actual retirement, there was an association with later retirement (3/3) (Thorsen et al. 2016; Kubicek et al. 2010; Mein et al. 2000). Of five papers that measured ‘challenge at work’, all found higher levels of challenge to be associated with later retirement intentions (4/4) (van den Berg 2011; Henkens and Leenders 2010; Henkens and Tazelaar 1997; Damman et al. 2011). High levels of challenge were also associated with later actual retirement (2/3) (Henkens and Tazelaar 1997; Damman et al. 2011). There were insufficient analyses of other subtypes to draw conclusions, but available evidence suggested that jobs that were meaningful (2/4) (Suadicani et al. 2013; Kilty and Behling 1985) or interesting (1/1) (Kilty and Behling 1985) may have encouraged later retirement.

**Table 4:** Direction of evidence for analyses of job satisfaction in relation to retirement timing

Measure of job satisfaction (number of papers that include this measure)	Analyses of retirement intentions				Analyses of actual retirement			
	Direction of evidence			Total analyses of retirement intentions	Direction of evidence			Total analyses of actual retirement
	Earlier retirement	Null	Later retirement		Earlier retirement	Null	Later retirement	
Job satisfaction (11)^a^	2	2	4	8	–	–	3	3
Challenge at work (5)	–	–	4	4	–	1	2	3
Meaningfulness of work (4)	–	–	2	2	–	2	–	2
Reward in work (2)	–	–	–	0	–	2	–	2
Job content plateau (1)^b^	1	–	–	1	–	–	–	0
Work providing active interest (1)	–	–	1	1	–	–	–	0
Total	3	2	11	16	0	5	5	10

^a^Includes ‘work enjoyment’ and ‘professional satisfaction’

^b^A measure of job dissatisfaction (the point at which a job becomes routine and boring)

#### Social support

There were 17 analyses of social support. Overall, the evidence was moderately in favour of the view that social support promotes later retirement. Social support was associated with later retirement in 6/11 analyses of intentions and 3/8 analyses of actual retirement. However, the measurement of social support varied considerably, and in most cases, only a few studies included the same measure (Supplementary Table 1). This makes it difficult to draw conclusions about subtypes of social support. Supervisor support was associated with intention to extend work in 2/2 (Ten Have et al. 2014; Oude Hengel et al. 2012) analyses, whereas support from colleagues was only related to retirement intentions in 1/3 (Oude Hengel et al. 2012) analyses, and in this study, it was associated with earlier retirement intentions.

#### Organisational resources

Six papers considered the influence of ‘organisational resources’ on retirement outcomes (Supplementary Table 6). For retirement intentions, 3/4 analyses found higher organisational resources to be associated with later retirement intentions (Hofstetter and Cohen 2014; van den Berg 2011; Heponiemi et al. 2008). There were two analyses of organisational resources in relation to actual retirement, one of which found greater organisational resources to be associated with later actual retirement (Thorsen et al. 2016). Organisational justice was the only subtype of organisational resources to be analysed in more than one paper. As most subtypes of organisational resources appeared only in a single paper, it is not possible to draw conclusions about subtypes of organisational resources.

#### Effort–reward imbalance (ERI)

Five papers considered effort–reward imbalance (see supplementary Tables 11 and 12). There were two analyses for retirement intentions, both of which found high ERI to be associated with earlier retirement intentions (Wahrendorf et al. 2013; Siegrist et al. 2007). Three analyses considered actual retirement, with just one finding high ERI to be associated with later actual retirement (Hintsa et al. 2015).

#### Job insecurity

Three papers considered job insecurity (see supplementary Tables 9 and 10). For retirement intentions, one analysis out of three found high job insecurity to be associated with intended earlier retirement (Stynen et al. 2016). There was only one analysis of job insecurity in relation to actual retirement, and it produced a null outcome (Lund and Villadsen 2005).

## Discussion

We performed a systematic review of evidence from 46 papers. There were sufficient analyses of job resources (38 papers), job demands (30 papers), job satisfaction (19 papers) and social support (17 papers) to draw conclusions about their effects on retirement. There was insufficient evidence about effort–reward imbalance (5 papers), job strain (1 paper) or job insecurity (3 papers) to draw conclusions about these workplace characteristics.

Overall, we found strong evidence to support the hypothesis that greater job resources are associated with later retirement intentions (23/37 analyses) and with later actual retirement (16/28 analyses). Job control was the most studied measure of job resource, with 13/22 and 10/18 analyses finding high job control to be associated with later retirement intentions and actual retirement, respectively. Just seven papers considered ‘opportunities to develop’ with mixed results. A possible explanation is that ‘opportunities for development’ may imply pressure to develop. For employees who are happy with their current level of development, the idea of further training might be daunting and may even precipitate earlier retirement. There was good evidence linking job satisfaction with later retirement outcomes, with 11/16 analyses linking satisfaction to later intended retirement and 5/10 linking it with later actual retirement. There was moderate evidence for the association between higher social support and extended working. There was less evidence regarding organisational resources and retirement (six papers). The majority of these focussed on intentions (4/6), meaning that there was insufficient evidence to draw any conclusions about organisational resources and actual retirement.

By contrast, there was limited evidence for the association of job demands with retirement. Most analyses found no association between higher job demands and retirement intentions (11/20 analyses) or actual retirement (18/22 analyses). Perceived job demands may influence retirement intentions without manifesting in actual retirement—for example if individuals are unable to retire due to financial circumstances. Another reason for the mixed findings could be that high demands are often associated with higher-status jobs, which tend also to be accompanied by higher job resources (Hintsa et al. 2015).

This review suggests that high-quality psychosocial working conditions may encourage later retirement. To retain older employees in the workforce, employers should seek to increase job resources, especially job control. This should include possibilities for flexible working, such as job sharing, self-rostering, working from home and split shifts (PRIME 2014). Flexible working might also help older workers with caregiving responsibilities. A greater emphasis on providing new learning opportunities tailored to older employees and greater valuing of the contribution of older employees would also promote extended working (de Wind et al. 2014).

### Strengths and limitations

To our knowledge, this is the first systematic review of the evidence on psychosocial work characteristics in relation to retirement. It drew upon a systematic review of high-quality studies, most published within the past decade. The recency of many of the studies included in our review may reflect how retirement and possibilities to extend working have become a more urgent policy and research priority, accompanying the recognition of an ageing population with fewer people in work to support those in retirement. In terms of limitations, the review was limited to non-disability retirement among employees who have not been identified as having ill health. Some of the included analyses were cross-sectional, and as such, participant characteristics could act as confounding variables that influence both the reporting of workplace psychosocial variables and the reporting of retirement intentions. Longitudinal studies of retirement behaviour demonstrate that perceived psychosocial factors at baseline are associated not only with retirement intentions but also with retirement behaviour at follow-up. This is consistent with psychosocial factors being causally implicated in retirement decisions. Given the considerable heterogeneity between papers regarding definitions of psychosocial factors and methods of statistical analysis, our review did not include a meta-analysis.

Another set of limitations arises from varying definitions of the retirement outcome in each study. For example, when some studies defined ‘retirement intentions’, they explicitly asked participants to ignore practical considerations (such as personal finances) whilst others did not. Some studies defined retirement behaviours in relation to a specific time point (e.g. statutory pension age), whereas others looked at the risk of stopping work, regardless of when this occurred.

### Future research

Future research should seek to incorporate more precise, and consistent, measures of psychosocial work characteristics. Within job resources, further research on ‘opportunities to develop’ would be valuable, especially to differentiate between optional skill development and compulsory training. With respect to job demands, there was some evidence that greater challenge at work promotes later retirement outcomes, but existing measures of demands probably conflate positive stimulating aspects of work with burdensome and effortful aspects of work. There was little evidence relating to organisational resources, but the included studies suggested an association between greater organisational resources and later retirement intentions. Future research should clarify the nature of organisational resources, based on standardised measures, and should examine associations with actual retirement, as existing studies are mostly limited to intentions.

### Conclusion

The results of this review have important implications for governments seeking to extend working life. Past studies have shown psychosocial work characteristics to be potentially modifiable (Egan et al. 2009; Bambra et al. 2007). It may be possible to prevent early exit from the workforce, before statutory pension age, by improving psychosocial work characteristics. This review found the following:

- There was strong evidence that a higher level of job resources, and specifically greater job control, is associated with later retirement (both intentions and actual retirement).
- There was very limited evidence that higher job demands are associated with earlier retirement intentions, and almost no studies found demands to be associated with actual retirement.

### Electronic supplementary material

Below is the link to the electronic supplementary material.

Supplementary material 1 (DOCX 117 kb)

## Appendix 1

### Search terms

- Psychosocial factors: job strain OR work demand* OR social support* OR work control OR decision latitude OR decision authority OR skill discretion OR effort reward OR work variety OR psychosocial work OR job insecurity.
- Retirement: resig* OR retir* OR retirement.
- N B All searches were filtered for English language studies and scientific articles.

The above searches were combined with AND.
