# Supplementary material for: The relationship between workplace psychosocial environment and retirement intentions and actual retirement: a systematic review
Source: Eur J Ageing. 2018 Apr 19;16(1):73–82. doi: 10.1007/s10433-018-0473-4 (PMC6397102; doi:10.1007/s10433-018-0473-4)
Supplement: Supplementary file 1 — Supplementary material 1 (DOCX 117 kb) [file 10433_2018_473_MOESM1_ESM.docx]

**Contents**

[Supplementary Table 1: Direction of evidence for analyses of social support in relation to retirement timing 3](#_Toc482197424)

[Supplementary Table 2: Direction of evidence for analyses of organisational resources in relation to retirement timing 4](#_Toc482197425)

[Supplementary Table 3: Evidence on job demands in relation to retirement intentions 5](#_Toc482197426)

[Supplementary Table 4: Evidence on job demands in relation to actual retirement 12](#_Toc482197427)

[Supplementary Table 5: Evidence on job resources in relation to retirement intentions 17](#_Toc482197428)

[Supplementary Table 6: Evidence on job resources in relation to actual retirement 25](#_Toc482197429)

[Supplementary Table 7: Evidence on work-based social support in relation to retirement intentions 31](#_Toc482197430)

[Supplementary Table 8: Evidence on work-based social support in relation to actual retirement 35](#_Toc482197431)

[Supplementary Table 9: Evidence on job insecurity in relation to retirement intentions 39](#_Toc482197432)

[Supplementary Table 10: Evidence on job insecurity in relation to actual retirement 40](#_Toc482197433)

[Supplementary Table 11: Evidence on effort-reward imbalance in relation to retirement intentions 41](#_Toc482197434)

[Supplementary Table 12: Evidence on effort-reward imbalance in relation to actual retirement 42](#_Toc482197435)

[Supplementary Table 13: Evidence on job satisfaction in relation to retirement intentions 44](#_Toc482197436)

[Supplementary Table 14: Evidence on job satisfaction in relation to actual retirement 50](#_Toc482197437)

[Supplementary Table 15: Evidence on organizational resources in relation to retirement intentions 54](#_Toc482197438)

[Supplementary Table 16: Evidence on organizational resources in relation to actual retirement 56](#_Toc482197439)

# **Supplementary Table 1:** Direction of evidence for analyses of social support in relation to retirement timing

| **Measure of social support**  (Number of papers that include this measure) | **Analyses of retirement intentions** | | | | **Analyses of actual retirement** | | | |
| --- | --- | --- | --- | --- | --- | --- | --- | --- |
|  | *Direction of evidence* | | | | *Direction of evidence* | | | |
|  | Early cessation of work | Null | Extended working | Total analyses of retirement intentions | Early cessation of work | Null | Extended working | Total analyses of actual retirement |
| Greater social support^†^ (6) | - | 1 | 1 | 2 | 1 | 2 | 1 | 4 |
| Greater co-worker support (3) | 1 | 2 | - | 3 | - | - | - | 0 |
| Greater supervisor support (2) | - | - | 2 | 2 | - | - | - | 0 |
| Higher quality of leadership at work (2) | - | - | 1 | 1 | - | 1 | - | 1 |
| Perceived pressure from colleagues to retire early^††^ (1) | - | 1 | - | 1 | - | 1 | - | 1 |
| Perceived supervisor support for working till 65 (1) | - | - | - | 0 | - | - | 1 | 1 |
| Conflicts in work (1) | - | - | - | 0 | 1 | - | - | 1 |
| Exposure to bullying (1) | 1 | - | - | 1 | - | - | - | 0 |
| Higher levels of team-working (1) | - | - | 1 | 1 | - | - | - | 0 |
| Total | 2 | 4 | 5 | 11 | 2 | 4 | 2 | 8 |
| *Notes.*  ^†^ This includes all studies which analysed social support without drawing more precise distinctions between different types of support. Where studies defined social support in more detail they appear on lower rows of the table.  ^††^ “I have the feeling that my colleagues will 'force' me to retire early”. | | | | | | | | |

# **Supplementary Table 2:** Direction of evidence for analyses of organisational resources in relation to retirement timing

| **Measure of organisational resource**  (Number of papers that include this measure) | **Analyses of retirement intentions** | | | | **Analyses of actual retirement** | | | |
| --- | --- | --- | --- | --- | --- | --- | --- | --- |
|  | *Direction of evidence* | | | Total analyses of retirement intentions | *Direction of evidence* | | | Total analyses of actual retirement |
|  | Early cessation of work | Null | Extended working |  | Early cessation of work | Null | Extended working |  |
| Higher perceived organisational support (1) | - | - | 1 | 1 | - | - | - | 0 |
| Greater organisational justice (2) | - | 1 | - | 1 | - | - | 1 | 1 |
| Higher organisational stimulation (1) | - | - | 1 | 1 | - | - | - | 0 |
| Greater organisational injustice (1) | 1 | - | - | 1 | - | - | - | 0 |
| Management quality (1) | - | - | - | 0 | - | 1 | - | 1 |
| **Total** | 1 | 1 | 3 | 5 | 0 | 1 | 1 | 2 |
|  | | | | | | | | |

# **Supplementary Table 3:** Evidence on job demands in relation to retirement intentions

| **Authors and Newcastle Ottawa Quality Assessment Grade (NOQAG)** | **Definition of psychosocial workplace characteristic** | **Retirement definition** | **Sample (population/database, N, gender balance, age range)** | **Follow-up** | **Response rate** | **Adjustments** | **Direction of evidence** |
| --- | --- | --- | --- | --- | --- | --- | --- |
| Stynen et al. (2016)  Cross-sectional NOQAG = 5  Longitudinal NOQAG = 6 | Psychological job demands (5 items)  Emotional demands (1 item) | “It is my intention to keep working until I reach the mandatory retirement age” | Maastricht Cohort Study (Netherlands population), N = 678, Male 90.9% female 9.1%, Age >50 (subdivided into 3 categories) | 2 years | 45% in the first year of data collection (1998). Response rate for 2012 unobtainable. | Emotional demands, gender, strenuous work, household situation, education level, economic sector | Cross sectional findings  Psychological job demands: NS Emotional job demands: NS  Longitudinal findings  Psychological job demands: NS Emotional job demands: NS |
| Leijten et al. (2015)  NOQAG = 5 | Psychological job demands (4 items) | Either self-report as ‘early retirement’ or retirement before the age of 65. | Dutch longitudinal Study on Transitions in Employment, Ability and Motivation, N = 8149, male 56.6% female 43.4%, age 45-64 | 3 years | 2010 = 71% response rate (baseline)  2011 = 82% of baseline  2012 = 80% of baseline  2013 = 74 % of baseline | Age, gender, educational level | Psychological job demands: NS |
| Frins et al. (2016)  NOQAG = 4 | Job demands (a single grouped analysis of the following variables: time pressure (2 items), task load (4 items based on JCQ), emotional demands (3 items from Copenhagen Psychosocial Questionnaire)) | Desired retirement age: ‘Until what age would you like to continue working?’ | Netherlands Working Conditions Cohort Study (NWCCS), N = 2897, Male 57% female 43%, age >50 | 1 year | 338% at baseline | None | Job demands at T1 were significantly related to desired retirement age at T2 (β = -0.05, p < 0.05) |
| Carr et al. (2016)  NOQAG = 7 | Psychosocial demands (2 items) | “At what age would you like to retire?” | English Longitudinal Study of Ageing, N = 3462, male 48.8% female 51.2%, age 50-69 | 10 years (analysis of how psychosocial conditions at T1 influenced preferences at T2) | 56% of participants from wave 1 responded to all subsequent waves | Age, self-rated health, income decile, long term health problems, disability, partner’s employment status | Higher psychosocial demands were associated with desire to retire earlier: β -0.18 (-0.31 to -0.08, p <0.01) |
| Burnay (2008)  NOQAG = 3 | Subjective stress: none/slight/average to a lot | ‘Ideally, at what age would you like to retire?’ (early retirement was defined as retirement aged <60) | P: Belgium; workers from 12 different industry sectors  N = 812  M:F = 53.6% male, 46.4% female  Age: all ages | N/A | Not reported | Sex, education level, no. of children, presence of spouse, housing type, place of residence. | OR of willingness to retire before age 60:  No stress = 1  Slight = 2.16 (p <0.05)  Average to a lot = 3.46 (p <0.05) |
| Sutinen et al. (2005)  NOQAG = 4 | Feeling overloaded (4 items) | Retirement preference: ‘If it were possible to choose between work and retirement, what would you choose?’ | P: Finland; postal questionnaire sent to physicians working in three hospital districts between 1997 and 1998.  N = 447  M:F = 56.2% male, 43.8% female  Age: all ages (26-63) | N/A | 55% | Age, sex, salary, minor psychiatric morbidity. | Feeling overloaded: NS |
| Zappala et al. (2008)  NOQAG = 3 | Job demands (demands and control were evaluated using 17 items) | Preference for early retirement (preferred retirement age lower than expected retirement age) | P: Italy; workers from public and private sectors.  N = 218  M:F = 45% male, 55% female  Age: 45-63 | N/A | Not reported | Age, sex, annual income, health, adequacy of financial situation for retirement, negative attitude to retirement, fear of social isolation, future planning, informed on pensions. | Job demands: NS |
| Harkonmaki et al. (2006)  NOQAG = 3 | Job demands based on the Karasek model (10 items). Scores divided into quartiles. | ‘Have you considered retiring before normal retirement age?’  (1=No intentions, 2=Weak intentions, 3=Strong intentions). | P: Finland, Helsinki Health Study (a postal questionnaire).  N = 5829  M:F = 19% male, 81% female  Age: 40-60 | N/A | 66% | Model 1: Age, socioeconomic status, physical health functioning, limiting long-standing illness, mental health status, job demands, procedural and relational justice.  Model 5: all model 1 controls plus procedural and relational justice, work-family and family-work conflicts, social network size. | High job demands were associated with increased probability of strong intentions to retire early (OR = 2.70; 95% C.I. 2.00-3.65). This became non-significant when additional controls were added in model 5 (OR = 1.38; C.I. 0.97-1.97). |
| Oude Hengel et al. (2011)  NOQAG = 3 | Quantitative job demands (4 items)  Emotional job demands (3 items) | Willingness to work until age 65. | P: Netherlands; construction workers (Netherlands Working Conditions Surveys)  N = 5610  M:F = 100% male  Age: 30-51 | N/A | 28.1% | Age, partnership status (yes, yes with a paid job, no), shiftwork, overtime work, dangerous work, musculoskeletal symptoms. | Quantitative demands: NS  Emotional demands: NS |
| Sejbaek et al. (2012)  NOQAG = 4 | Job demand (4 items) | Retirement intentions:  Normal (65 years)  Early (62-64 years)  Very early (≤61 years). | P: Denmark; employees in the elder care sector  N = 2444  M:F = 4% male, 96% female  Age: 45-57 (at T1) | Average of 24 months | 64.0% (T1)  60.5% (T2) | Sex, age, seniority, marital status, working schedule (day/shift work) type of occupation. | Job demand: NS |
| Suadicani et al. (2013)  NOQAG = 3 | Quantitative job demands (4 items)  Emotional job demands (3 items) | Intention to quit: ‘If I had the economic opportunity to do so, I would quit my job’ (yes/no) | P: Denmark; permanent staff in Bispebjerg University Hospital  N = 1809  M:F = 4% male, 96% female  Age: 45-57 (at T1) | N/A | 65% | Age, sex. | Quantitative demands: 2 out of 4 items were significant. One was associated with increased risk of early retirement intentions, whereas the other was associated with decreased risk of early retirement intentions.  Emotional demands: 2 out of 3 items were significantly associated with increased likelihood of early retirement intentions. |
| Elovainio et al. (2005)  NOQAG = 3 | Job demands (4 items) | Early retirement thoughts (2 items):  (1) 'Do you think that you are able to cope with your work until your official retirement age?'  (2) 'Have you considered seeking disability pension, individual early retirement pension or other form of pension?' | P: Finland, all employees in health and social services sector  N = 3072  M:F = 9% male, 91% female  Age: 20-65 | N/A | 61.4% | Age, gender, marital status, education level, self-rated health. | Higher job demands were associated with increased early retirement thoughts: OR = 1.41 (C.I. 1.29-1.54). |
| Henkens & Leenders (2010)  NOQAG = 4 | Workload (4 items) | Retirement intention (4 items) | P: Netherlands, survey of four private companies and one organisation of the Dutch government  N = 2892  M:F = 76% male, 24% female  Age: 50+ | N/A | 63% | Age, sex, education, sector (public/private), tenure (full time/part time), position (executive/non-executive), replacement rate. | High workload is associated with increased intention to retire early (β = 0.13, p <0.001).  When adjusted for burnout workload is not significantly associated with early retirement intentions. |
| Kilty & Behling (1985)  NOQAG = 5 | Work is too consuming (3 items) | Age at which participants plan to retire (1 item)  Whether participants have considered early retirement (1 item) | P: America; a sample of lawyers, social workers, college professors and high school teachers from Ohio  N = 457  M:F = 52% male, 48% female  Age: 25-64 | N/A | Not reported | Age, sex, profession (attorney, social worker, high school teacher), years of education, marital status, have a dependent child, self-employed, number of jobs, timing current position, household income, hours working. | Considered early retirement?  Work is too consuming: NS  Age of planned retirement  Work is too consuming: NS |
| Oakman & Wells (2012)  NOQAG = 3 | Job demands (12 items) | What is your intended timing of retirement? (within the next 5 years or beyond the next 5 years). | P: Australia; questionnaires distributed via the intranet in a large public services company in Victoria.  N = 332  M:F = 61% male, 39% female  Age: All ages (average 43.5) | N/A | 47.4% | Age, gender, length of service, marital status, dependent children. | Job demands: NS |
| Schreurs et al. (2011a)  NOQAG = 4 | Job demands: workload (3 items) and problems with change at work (3 items). | Early retirement intention (4 items). Early retirement was defined as retirement before the country’s official retirement age. | P: Belgium; stratified sample from large online panel of Belgian active working population  N = 1812  M:F = 62% male, 38% female  Age: >45 (average age of 48) | N/A | 38.8% | Sex, education, occupational status (stratified for age). | High job demands were associated with increased intention to retire early (γ = -0.32; p < 0.001). |
| Ten Have et al. (2014)  NOQAG = 6 | High psychological job demands (assessed using the Job Content Questionnaire) | Intention to work beyond retirement age: ‘If it was up to you, do you want to continue working after the age of 65 years?’ | P: Netherlands Mental Health Survey and Incidence Study-2  N = 1854  M:F = unclear  Age: 50-59 (at baseline) | N/A | 65.1% | Gender, age, education, partner status, having children, income situation | High psychological job demands: NS |

(NS)= >0.05, (*)= <0.05, (**)= <0.001

# **Supplementary Table 4:** Evidence on job demands in relation to actual retirement

| **Authors and Newcastle Ottawa Quality Assessment Grade (NOQAG)** | **Definition of psychosocial workplace characteristic** | **Retirement definition** | **Sample (population/database, N, gender balance, age range)** | **Follow up** | **Response rate** | **Adjustments** | **Direction of evidence** |
| --- | --- | --- | --- | --- | --- | --- | --- |
| Thorsen et al. (2016)  NOQAG = 8 | Work pace  Quantitative demands  Emotional demands  Role conflicts | Retirement age of 60-64 years as recorded in the Danish national register of social welfare beneficiaries. | DANES 2008 questionnaire survey, N = 1876, 50% male and 50% female, age 60-64 | 4 years | 66%, 69%, 77% (sample was drawn from 3 separate questionnaires) | Physical strain, socioeconomic status, gender, cohabitation, shift and night work, part-time work, sample, data collection, mental health, self-rated health | Work pace: NS  Quantitative demands: NS  Emotional demands: NS  Role conflicts: NS |
| Carr et al. (2016)  NOQAG = 7 | Psychosocial demands (2 items) | Actual exit from work was a shift from >0 hours of work per week to 0 hours of work per week (between two waves of data collection) | English Longitudinal Study of Ageing, N = 3462, male 48.8% female 51.2%, age 50-69 | 10 years | 56% of participants from wave 1 responded to all subsequent waves | Age, self-rated health, income decile, long term health problems, disability, partner’s employment status | Psychosocial demands: NS |
| Blekesaune & Solem (2005)  NOQAG = 9 | Average job stress for each job type based on surveys in 1987, 1991 and 1995. Male and female levels were calculated separately. | A drop in work-related income from >$12,000 to <$9000 | P: Norwegian; survey data (measuring working conditions) linked to administrative and census data  N = 19,114  M:F = 56% male, 44% female  Age: 60-66 | Follow up period = 7 years  Average individual follow up = 3.4 years | Not reported | Age, education, marital status, previous income, and “being above the eligible age for AFP” (AFP is a negotiated early pension scheme).  Separate models for men and women women | High job stress was associated with later non-disability retirement in men (-0.11; p < 0.05). The association was NS in women. |
| Kubicek et al. (2010)  NOQAG = 6 | Psychosocial job demands (2 items) | Self-reported actual retirement (telephone interview). Early retirement is retirement at or before age 62. | P: USA (Wisconsin Longitudinal Study); a random sample of graduates of Wisconsin high schools in 1957, participants had to be married at baseline and follow up, and they had to have worked for at least 10 years  N = 2499  M:F = 55.7% male, 44.3% female  Age: 51-56 at baseline | 11 years | Not reported | Pension plan, health insurance, spouses pension plan, spouse’s health insurance, educational attainment, income, occupational education, marital satisfaction (a factor comprised of family-to-work conflict, family resources, and family demands). | Higher psychosocial job demands were indirectly associated with increased early retirement. However, there was no direct association. |
| Robroek et al. (2013)  NOQAG = 7 | Time pressure (1 item): ‘I am under constant time pressure due to a heavy workload’. | Early retirement = self-reported retirement before the statutory country-specific retirement age. | P: 11 European countries (SHARE dataset)  N = 4923  M:F = 56.6% male, 43.4% female  Age: >50 (average age of 55.2) | 4 years | 62% (household response rate) | Age, sex, education, cohabitation status, health, health behaviour, lack of physical activity, smoking, alcohol intake. | Time pressure: NS |
| Clausen et al. (2014)  NOQAG = 4 | Role conflicts (4 items)  Emotional demands (4 items) | Self-reported work status: still employed/quit/retired | P: Denmark; employees from Danish Eldercare services in 35 municipalities.  N = 7025  M:F = 4% male, 96% female  Age: all ages | 18-24 months | 78% at baseline, 73.5% at follow up | None | Early retirees experienced significantly less role conflict than those who kept working (p <0.01)  Emotional demands: NS |
| Lund et al. (2001)  NOQAG = 6 | Job demands (derived from a 20 item ‘psychosocial work environment’ scale). | Self-reported receipt of early retirement pension. | P: Denmark; a sample of waste collectors and municipal workers  N = 2618  M:F = 100% male  Age: all ages | 2.5 years | Waste collectors = 75.9%  Municipal workers = 81.7% (at baseline) | Age, occupational group, health, smoking, marital status. | Job demands: NS |
| Mein et al. (2000)  NOQAG = 6 | Job demands (4 items) measured in the ‘Karasek Job Context Instrument’ | Retirement before age 59.5 years. | P: UK; London based civil servants (Whitehall II Study)  N = 2532  M:F = 67% male, 33% female  Age: 50-59.5 | 7 years | 73% | Age, duration of employment in civil service, grade, job satisfaction, perceived health, marital status, material problems, housing, car access, long term illness, general health, job demands, work support. | Job demands  Men: NS  Women: NS |
| Boot et al. (2014)  NOQAG = 8 | Psychosocial demands (derived from a job exposure matrix). Included task requirements, time pressure, cognitive demands. | Having <1 hour of paid work per week at follow up. | P: Netherlands  N = 333  M:F = 58.6% male; 41.4% female  Age: 55-65 | 3 years | 55% at baseline, 95% at follow up | Gender, age, education, partnership status, partner employment status, satisfaction with income level, satisfaction with living standard, self-rated health, depressive symptoms, functional limitations, mastery, self-esteem, neuroticism and social inadequacy scales, working hours, "occupational prestige" and "occupational skill level". | Psychosocial demands: NS |
| Donbaek-Jensen et al. (2012)  NOQAG = 5 | Job demands based on Karasek’s Job Content Questionnaire (3 items) | Permanent voluntary early retirement as recorded in registry data. | P: Denmark; all nurses’ aides in Aarhus  N = 3332  M:F = 2% male; 98% female  Age: all ages | 15 years | 74% | ‘Relevant covariates tested for collinearity’ – collinearity was not found. | Increased job demands were associated with increased hazard ratio for voluntary early retirement (HR = 1.28; C.I. 1.09-1.50). |
| Friis et al. (2007)  NOQAG = 7 | Pressure of work: ‘How often are you so busy that you have difficulty in completing your tasks at work?’  Busyness at work: ‘What is the pressure or tempo of your work?’ | Exit from the labour market into Post Employment Wage (PEW), a form of early retirement payment/pension. | P: Denmark, all the female members of the Danish nurses association  N = 5538  M:F = 0% male; 100% female  Age: 51-59 | 9 years | 86% | Self-reported health, marital status, spouse's social economic status and income, annual gross income, place of residence, leisure time physical activity, number of drinks over that latest weekend, smoking behaviour, body mass index (BMI). | High pressure of work was associated with increased early retirement (HR 1.09; 95% C.I. 1.01-1.17).  Busyness at work: NS |
| Van Solinge & Henkens (2013)  NOQAG = 3 | Job pressure (3 items) | Actual retirement age as established by survey. | P: Netherlands; employees of civil service and 3 large private companies  N = 1460  M:F = unclear  Age: 50-59 (at baseline) | 10 years | 62% | Partner work status, wealth, income and health, organisation. | Job pressure: NS |
| Robroek et al. (2015)  NOQAG = 7 | Job demands (2 items) | Early retirement (before age 65 years) receiving pre-pension as main income | P: Netherlands; ‘Permanent Survey on Living Conditions’  N = 2922  M:F = 58.8% male; 41.2% female  Age: 50-64 | 10 years | Original response rate 60-65%. Questionnaire data linked to registry data with passive consent. | Sex, age, marital status, education, health status, smoking, heavy alcohol intake, BMI, level of sporting activity. | Job demands: NS |
| Lund & Villadsen (2005)  NOQAG = 8 | Emotional demands (3 items), demands of bottling up emotions (2 items). | Receipt of ERP (Early Retirement Pension) during 01/01/2001 to 31/12/2014 | P: Denmark (DWECS/DREAM database)  N = 365  M:F = 54.8% male, 45.2% female  Age: 57-62 | 4 years | Not reported | Gender, age, cohabitation, social economic position, physical demands. | Emotional demands: NS  Demands of bottling up emotions: NS |
| Virtanen et al. (2014)  NOQAG = 8 | Job strain: ‘difference between job demands and job control’ | Remaining in employment 6 months beyond pensionable age | P: Finland; employees working in 10 towns and 21 hospitals.  N = 4677  M:F = 27.5% male, 72.5% female  Age: those who reach pensionable age between 2005-2011 | Mean = 3.7 years | 72% | Individual pensionable age, sex, socioeconomic status, marital status, residence and area, type of employment contract, work schedule, part-time pension, job strain, effort-reward imbalance, work time control, chronic disease, symptoms of ill health, smoking, alcohol use, leisure time physical activity, and obesity | Job strain: NS |

(NS)= >0.05, (*)= <0.05, (**)= <0.001

# **Supplementary Table 5:** Evidence on job resources in relation to retirement intentions

| **Authors and Newcastle Ottawa Quality Assessment Grade (NOQAG)** | **Definition of psychosocial workplace characteristic** | **Retirement definition** | **Sample (population/database, N, gender balance, age range)** | **Follow up** | **Response rate** | **Adjustments** | **Direction of evidence** |
| --- | --- | --- | --- | --- | --- | --- | --- |
| Stynen et al. (2016)  Cross-sectional NOQAG = 5  Longitudinal NOQAG = 6 | Availability of training (2 items)  Availability of job enrichment/rotation (2 items)  Availability of career development (2 items)  Flexible working (3 items)  Availability of workplace adaptations (3 items)  Decision latitude (from JCQ) | “It is my intention to keep working until I reach the mandatory retirement age” | Maastricht Cohort Study (Netherlands population), N = 678, Male 90.9% female 9.1%, Age >50 (subdivided into 3 categories) | 2 years | 45% in the first year of data collection (1998). Response rate for 2012 unobtainable. | Emotional demands, gender, strenuous work, household situation, education level, economic sector | Longitudinal findings  Age 55-59:  Availability of training: (β = 0.13. S.E. = 0.10, p <0.05)  Availability of job enrichment/rotation: (β = 0.13. S.E. = 0.08, p <0.05)  Availability of career development: (β = 0.14. S.E. = 0.08, p <0.05)  Decision latitude: (β = 0.14. S.E. = 0.01, p <0.05)  All other variables: NS |
| Frins et al. (2016)  NOQAG = 4 | Job resources (the variables listed below were all analysed together in a single analysis)  Autonomy (5 items from JCQ)  Task variety (3 items from JCQ)  Social support from supervisor (4 items from JCQ)  Social support from colleagues (4 items from JCQ) | Desired retirement age: ‘Until what age would you like to continue working?’ | Netherlands Working Conditions Cohort Study (NWCCS), N = 2897, Male 57% female 43%, age >50 | 1 year | Should be obtainable from another article on NWCCS | None | Job resources at T1 were significantly related to desired retirement age at T2 (β = 0.11, p < 0.01) |
| Carr et al. (2016)  NOQAG = 7 | Decision authority (2 items)  Recognition (1 item) | “At what age would you like to retire?” | English Longitudinal Study of Ageing, N = 3462, male 48.8% female 51.2%, age 50-69 | 10 years | 56% of participants from wave 1 responded to all subsequent waves | Age, self-rated health, income decile, long term health problems, disability, partner’s employment status | Decision authority was associated with desire to retire later: β 0.38 (0.23 to 0.53, p <0.001)  Recognition: NS |
| Sutinen et al. (2005)  NOQAG = 4 | Job control from the Job Content Questionnaire (9 items) | Retirement preference: ‘If it were possible to choose between work and retirement, what would you choose?’ | P: Finland; postal questionnaire sent to physicians working in three hospital districts between 1997 and 1998.  N = 447  M:F = 56.2% male, 43.8% female  Age: all ages (26-63) | N/A | 55% | Age, sex, salary, minor psychiatric morbidity. | Low job control is associated with increased retirement preference (β value = -0.22). |
| Van den Berg (2011)  NOQAG = 3 | Job autonomy (5 items)  Work variety (4 items) | Willingness to continue working (2 items) | P: Netherlands; participants recruited while attending training classes at a professional knowledge centre.  N = 73  M:F = 52% male, 48% female  Age: 50-65 | N/A | 45% | Sex, age, job tenure | High job autonomy was significantly associated with willingness to continue working (β = 0.40; C.I. 0.22-0.28, p < 0.05)  High work variety was significantly associated with willingness to continue working (β = 0.24; C.I. 0.05-0.43, p < 0.05) |
| Siegrist et al. (2006)  NOQAG = 6 | Job control (2 items) | Intended retirement: ‘Thinking about your present job, would you like to retire as early as possible?’ [yes/no] | P: 10 European countries (SHARE dataset)  N = 6836  M:F = 51.5% male, 48.5% female  Age: from 50-65 | N/A | 55.4% (household response rate) | Country, gender, age, education, income, self-perceived health, depressive symptoms, physical symptoms, quality of life in old age. | Low job control was significantly associated with desire to retire as early as possible (OR = 1.51*; C.I. 1.27-1.80). |
| Zappala et al. (2008)  NOQAG = 3 | Job control (demands and control were evaluated using 17 items)  Opportunity to develop abilities (6 items) | Preference for early retirement (preferred retirement age lower than expected retirement age) | P: Italy; workers from public and private sectors.  N = 218  M:F = 45% male, 55% female  Age: 45-63 | N/A | Not reported | Age, sex, annual income, health, adequacy of financial situation for retirement, negative attitude to retirement, fear of social isolation, future planning, informed on pensions. | Job control: NS  Increased opportunity to develop abilities was associated with an increased preference for early retirement (β = -0.16; p <0.05). |
| Harkonmaki et al. (2006)  NOQAG = 3 | Job control based on the Karasek model (9 items). Scores divided into quartiles. | ‘Have you considered retiring before normal retirement age?’ (1=No intentions, 2=Weak intentions, 3=Strong intentions). | P: Finland, Helsinki Health Study (a postal questionnaire).  N = 5829  M:F = 19% male, 81% female  Age: 40-60 | N/A | 66% | Model 1: Age, socioeconomic status, physical health functioning, limiting long-standing illness, mental health status, job demands, procedural and relational justice.  Model 5: all model 1 controls plus procedural and relational justice, work-family and family-work conflicts, social network size. | Low job control was associated with strong intentions to retire early (OR = 1.95*; 85% C.I. 1.45-2.61). This remained significant when additional controls were added in model 5 (OR = 1.49*; C.I. 1.07-2.08). |
| Heponiemi et al. (2008)  NOQAG = 3 | Job control composed of skill discretion (6 items) and decision authority (3 items) | Strength of retirement intentions (3 levels) based on two items | P: Finland, physicians drawn from the database of the Finnish Medical Association  N = 1383  M:F = 51% male, 49% female  Age: 45-65 | N/A | 57% | Age, sex, employment sector (health centre, hospital, other), self-rated health, sickness absence within 12 months. | Low job control was associated with increased intentions to retire early (OR = 1.71*; C.I. 1.50-1.95) |
| Oude Hengel et al. (2011)  NOQAG = 3 | Skill discretion  Job autonomy | Willingness to work until age 65. | P: Netherlands; construction workers (Netherlands Working Conditions Surveys)  N = 5610  M:F = 100% male  Age: 30-51 | N/A | 28.1% | Age, partnership status (yes, yes with a paid job, no), shiftwork, overtime work, dangerous work, musculoskeletal symptoms. | Skill discretion: intermediate skill discretion was associated with decreased willingness to work to 65 (0.79; C.I. 0.66-0.94) but low and high skill discretion were not significant.  Job autonomy: NS |
| Sejbaek et al. (2012)  NOQAG = 4 | Job resources (4 items) | Retirement intentions:  Normal (65 years)  Early (62-64 years)  Very early (≤61 years). | P: Denmark; permanent staff in Bispebjerg University Hospital  N = 1809  M:F = 4% male, 96% female  Age: 45-57 (at T1) | Average 24 months | 64.0% (T1)  60.5% (T2) | Sex, age, seniority, marital status, working schedule (day/shift work) type of occupation. | Job resources: NS |
| Suadicani et al. (2013)  NOQAG = 3 | Decision latitude  (3 items) | Intention to quit: ‘If I had the economic opportunity to do so, I would quit my job’ (yes/no) | P: Denmark  N = 1809  M:F = 4% male, 96% female  Age: 45-57 (at T1) | N/A | 65% | Age, sex. | Low decision latitude was associated with intentions to quit job. |
| Thorsen et al. (2012)  NOQAG = 4 | Recognition (1 item)  Influence (2 items)  Possibilities for development (2 items)  Lack of predictability (2 items) | Intention to retire before age 65. | P: Denmark; Danish National working Environment Study, an internet survey.  N = 3122  M:F = 47% male, 53% female  Age: >50 | N/A | 77% | Separate analyses  Gender, age,  vocational education, physical  work strain, occupational group,  socio-economic status, data collection mode, self-rated health,  depressive symptoms,  work performance  and work ability.  Combined analyses  As above, but the analysis of each variable included all covariates and the four significant psychosocial variables (ageism, lack of possibilities for development, lack of recognition, lack of predictability). | Lack of possibilities for development (OR = 1.11; C.I. 1.01-1.22, p = 0.04)  Lack of recognition: NS  Lack of predictability: NS  Low influence: NS |
| Von Bonsdorff et al. (2010)  NOQAG = 3 | Job control (8 items) | ‘Have you ever considered retiring before full retirement age?’ [Full retirement age in Finland has varied between 63-68 years since 2005] | P: Finland; employees from the metal industry and retail trade.  N = 1281  M:F = metal industry 78% male, 22% female; retail trade 15% male, 85% female.  Age: ≥45 | N/A | 25.5% of managers  54.5% of employees (from the companies whose managers responded) | Age, sex, marital status, field of industry | High control was related to having weak rather than strong early retirement intentions (OR = 1.58; C.I. 1.13-2.21; p <0.01) |
| Elovainio et al. (2005)  NOQAG = 3 | Job control: ‘extent to which employees felt free to determine the quantitative and qualitative factors of their own work process’ (3 items) | Early retirement thoughts (2 items):  (1) 'Do you think that you are able to cope with your work until your official retirement age?'  (2) 'Have you considered seeking disability pension, individual early retirement pension or other form of pension?' | P: Finland, all employees in health and social services sector  N = 3072  M:F = 9% male, 91% female  Age: 20-65 | N/A | 61.4% | Age, gender, marital status, education level, self-rated health. | Higher job control was associated with decreased early retirement thoughts: OR = 0.8* (C.I. 0.73-0.88). |
| Henkens & Leenders (2010)  NOQAG = 4 | Autonomy (1 item)  Opportunities for growth (2 items) | Retirement intention (4 items) | P: Netherlands, survey of four private companies and one organisation of the Dutch government  N = 2892  M:F = 76% male, 24% female  Age: 50+ | N/A | 63% | Age, sex, education, sector (public/private), tenure (full time/part time), position (executive/non-executive), replacement rate. | Autonomy: NS  Higher opportunities for growth were associated with a decreased intention to retire early (β = -0.07, p <0.001). |
| Kilty & Behling (1985)  NOQAG = 5 | Work related autonomy (4 items) | Age at which participants plan to retire (1 item)  Whether participants have considered early retirement (1 item) | P: America; a sample of lawyers, social workers, college professors and high school teachers from Ohio  N = 457  M:F = 52% male, 48% female  Age: 25-64 | N/A | Not reported | Age, sex, profession (attorney, social worker, high school teacher), years of education, marital status, have a dependent child, self-employed, number of jobs, timing current position, household income, hours working. | Considered early retirement?  Work related autonomy: NS  Age of planned retirement  Work related autonomy: NS |
| Munderlein & Koster (2013)  NOQAG = 4 | Autonomy (4 items) | Intention to retire in the next 12 months | P: Netherlands; STREAM survey (Study on Transitions in the Employment, Ability and Motivation), a longitudinal survey using an internet panel.  N = 10,849  M:F = 57% male, 43% female  Age: 45-64 | N/A | 71% | Age, sex, general health, education, sufficient income, temporary employment, part-time employment, tenure position, supervisory status, industry. | Autonomy: NS |
| Oakman & Wells (2012)  NOQAG = 3 | Social cohesion (9 items)  Job control (7 items) | What is your intended timing of retirement? (within the next 5 years or beyond the next 5 years). | P: Australia; questionnaires distributed via the intranet in a large public services company in Victoria.  N = 332  M:F = 61% male, 39% female  Age: All ages (average 43.5) | N/A | 47.4% | Age, gender, length of service, marital status, dependent children. | Higher social cohesion was associated with an increased intention to retire within the next 5 years (OR = 1.46; C.I. 1.02-2.10)  Job control: NS |
| Wahrendorf et al. (2013)  NOQAG = 4 | Job control (2 items) | Early retirement intentions “Thinking about your current job, would you like to retire as early as possible?” [Yes/No] | P: 11 European countries (SHARE data)  N = 6398  M:F = 55.6% male, 44.4% female  Age: 50-64 | N/A | 60.6% | Gender, age, functional limitations, country, work stress | Low work control was associated with increased early retirement intentions (OR = 1.60; p <0.001) |
| Ten Have et al. (2014)  NOQAG = 6 | Low decision latitude  (assessed using the Job Content Questionnaire) | Intention to work beyond retirement age: ‘If it was up to you, do you want to continue working after the age of 65 years?’ | P: Netherlands Mental Health Survey and Incidence Study-2  N = 1854  M:F = unclear  Age: 50-59 (at baseline) | N/A | 65.1% | Gender, age, education, partner status, having children, income situation | Low decision latitude was associated with decreased intention to work beyond age 65: OR=0.70 (95% C.I. 0.53-0.92) |

(NS)= >0.05, (*)= <0.05, (**)= <0.001

# **Supplementary Table 6:** Evidence on job resources in relation to actual retirement

| **Authors and Newcastle Ottawa Quality Assessment Grade (NOQAG)** | **Definition of psychosocial workplace characteristic** | **Retirement definition** | **Sample (population/database, N, gender balance, age range)** | **Follow up** | **Response rate** | **Adjustments** | **Direction of evidence** |
| --- | --- | --- | --- | --- | --- | --- | --- |
| Thorsen et al. (2016)  NOQAG = 8 | Low influence  Low possibilities of development  Poor predictability | Retirement age of 60-64 years as recorded in the Danish national register of social welfare beneficiaries. | DANES 2008 questionnaire survey, N = 1876, 50% male and 50% female, age 60-64 | 4 years | 66%, 69%, 77% (sample was drawn from 3 separate questionnaires) | Physical strain, socioeconomic status, gender, cohabitation, shift and night work, part-time work, sample, data collection, mental health, self-rated health | Low influence was associated with increased hazard ratio for early retirement HR **=** 1.56 (C.I. 1.16-2.09; p = 0.003)  Low possibilities of development were associated with increased hazard ratio for early retirement: HR = 2.04 (C.I. 1.40-2.98; p = 0.0002)  Poor predictability was associated with increased hazard ratio for early retirement: HR = 1.42 (C.I. 1.01-2.00; p = 0.04) |
| Leijten et al. (2015)  NOQAG = 5 | Autonomy (5 items) | Either self-report as ‘early retirement’ or retirement before the age of 65. | Dutch longitudinal Study on Transitions in Employment, Ability and Motivation, N = 8149, male 56.6% female 43.4%, age 45-64 | 3 years | 2010 = 71% response rate (baseline)  2011 = 82% of baseline  2012 = 80% of baseline  2013 = 74 % of baseline | Age, gender, educational level | Autonomy: NS |
| Carr et al. (2016)  NOQAG = 7 | Decision authority (2 items)  Recognition (1 item) | Actual exit from work was a shift from >0 hours of work per week to 0 hours of work per week (between two waves of data collection) | English Longitudinal Study of Ageing, N = 3462, male 48.8% female 51.2%, age 50-69 | 10 years | 56% of participants from wave 1 responded to all subsequent waves | Age, self-rated health, income decile, long term health problems, disability, partner’s employment status | High decision authority was associated with decreased odds ratio of actual retirement: OR = 0.93 (0.88 to 0.97, p <0.05)  Low recognition was associated with increased odds ratio of actual retirement: OR = 1.23 (1.10 to 1.43, p <0.01) |
| Blekesaune & Solem (2005)  NOQAG = 9 | Average job autonomy for each job type based on surveys in 1987, 1991 and 1995. Male and female autonomy levels were calculated separately. | A drop in work-related income from >$12,000 to <$9000 | P: Norwegian; survey data (measuring working conditions) linked to administrative and census data  N = 19,114  M:F = 56% male, 44% female  Age: 60-66 | Follow up period = 7 years  Average individual follow up = 3.4 years | Not reported | Age, education, marital status, previous income, and “being above the eligible age for AFP” (AFP is a negotiated early pension scheme).  Separate models for men and women | Low autonomy was associated with non-disability retirement in men (logit coefficient 0.16, p <0.01). The association was NS in women. |
| Robroek et al. (2013)  NOQAG = 7 | Job control (2 items) | Early retirement = self-reported retirement before the statutory country-specific retirement age. | P: 11 European countries (SHARE dataset)  N = 4923  M:F = 56.6% male, 43.4% female  Age: from 50 up to the country-specific retirement age | 4 years | 62% | Age, sex, education, cohabitation status, health, health behaviour, lack of physical activity, smoking, alcohol intake. | Low job control associated with early retirement (HR = 1.30; C.I. 1.08-1.57). |
| Kubicek et al. (2010)  NOQAG = 6 | Job resources (2 items) | Self-reported actual retirement (telephone interview). Early retirement is retirement at or before age 62. | P: USA (Wisconsin Longitudinal Study); a random sample of graduates of Wisconsin high schools in 1957, participants had to be married at baseline and follow up, and they had to have worked for at least 10 years  N = 2499  M:F = 55.7% male, 44.3% female  Age: 51-56 at baseline | 11 years | Not reported | Pension plan, health insurance, spouses pension plan, spouse’s health insurance, educational attainment, income, occupational education, marital satisfaction (a factor comprised of family-to-work conflict, family resources, and family demands). | Higher job resources are directly associated with decreased early retirement. No statistics are reported in the paper but the above is stated in the text. |
| Clausen et al. (2014)  NOQAG = 4 | Influence at work (4 items) | Self-reported work status: still employed/quit/retired | P: Denmark; employees from Danish Eldercare services in 35 municipalities.  N = 7025  M:F = 4% male, 96% female  Age: all ages | 18-24 months | 78% at baseline, 73.5% at follow up | None reported | Early retirees experienced significantly less influence at work than those who kept working (p <0.001). |
| Virtanen et al. (2014)  NOQAG = 8 | Work time control: control over start and end times, breaks, scheduling of shifts and total working hours. | Remaining in employment 6 months beyond pensionable age | P: Finland; employees working in 10 towns and 21 hospitals.  N = 4677  M:F = 27.5% male, 72.5% female  Age: those who reach pensionable age between 2005-2011 | Mean = 3.7 years | 72% | Individual pensionable age, sex, socioeconomic status, marital status, residence and area, type of employment contract, work schedule, part-time pension, job strain, effort-reward imbalance, work time control, chronic disease, symptoms of ill health, smoking, alcohol use, leisure time physical activity, and obesity | Increased work time control was associated with remaining in employment 6 months beyond retirement age (Low control = 1.00  Average control = 1.36; C.I. 1.10-1.68  High control = 2.31; C.I. 1.88-2.84) |
| Lund et al. (2001)  NOQAG = 6 | Decision authority and skill discretion (derived from a 20 item ‘psychosocial work environment’ scale). | Self-reported receipt of early retirement pension. | P: Denmark; a sample of waste collectors and municipal workers  N = 2618  M:F = 100% male  Age: all ages | 2.5 years | 78.8% | Age, occupational group, health, smoking, marital status. | Decision authority: NS  Skill discretion: NS |
| Donbaek-Jensen et al. (2012)  NOQAG = 5 | Decision latitude based on Karasek’s Job Content Questionnaire (3 items) | Permanent voluntary early retirement as recorded in registry data. | P: Denmark; all nurses’ aides in Aarhus  N = 3332  M:F = 2% male; 98% female  Age: all ages | 15 years | 74.4% | ‘Relevant covariates tested for collinearity’ – collinearity was not found. | Decision latitude: NS |
| Friis et al. (2007)  NOQAG = 7 | Influence at work: ‘How much influence do you have on the organisation of your daily work?’ | Exit from the labour market into Post Employment Wage (PEW), a form of early retirement payment/pension. | P: Denmark, all the female members of the Danish nurses association  N = 5538  M:F = 0% male; 100% female  Age: 51-59 | 9 years | 86% | Self-reported health, marital status, spouse's social economic status and income, annual gross income, place of residence, leisure time physical activity, number of drinks over that latest weekend, smoking behaviour, body mass index (BMI). | Lower influence at work was associated with increased early retirement (HR 1.09; 95% C.I. 1.01-1.18). |
| Herrbach et al. (2009)  NOQAG = 5 | Access to training  Flexibility of working hours  Opportunity for role change | Actual voluntary early retirement | P: France; graduates of French universities between 1967 and 1977 employed in the private sector  N = 300  M:F = 92% male; 8% female  Age: 50+ | 30 months | 20.5% at follow up | Age, sex, organisational tenure, perceived personal health, perceived spouse’s health, work centrality. | Increased access to training was associated with decreased early retirement (OR = 0.44; p < 0.01)  Flexibility of working conditions: NS  Opportunity for role change was associated with increased early retirement (OR = 1.86; p < 0.05) |
| Van Solinge & Henkens (2013)  NOQAG = 3 | Growth/promotion opportunities (3 items)  Perceived schooling/training opportunities (1 item)  Flexibility of work time and place (4 items) | Actual retirement age as established by survey. | P: Netherlands; employees of civil service and 3 large private companies  N = 1460  M:F = unclear  Age: 50-59 (at baseline) | 10 years | 62% | Partner work status, wealth, income and health, organisation. | More growth/promotion opportunities were associated with later retirement (HR = 0.943; p <0.05).  Perceived schooling/training opportunities: NS  Flexibility of work time and place: NS |
| Hintsa et al. (2015)  NOQAG = 7 | Job control measured using ‘a short battery of items derived from the Job Content Questionnaire’ | Exit from the labour market at or below age 61. | P: England; English Longitudinal Study of Aging (ELSA)  N = 1263  M:F = 52.3% male; 47.7% female  Age: 50-74 years | 6 years | Not given. | Age, sex, education, occupational class and allostatic load. | Lower job control is associated with early exit from the labour market: OR = 0.58; C.I. 0.39-0.85 (complete cases analysis) |
| Robroek et al. (2015)  NOQAG = 7 | Job control (5 items) | Early retirement (before age 65 years) receiving pre-pension as main income | P: Netherlands; ‘Permanent Survey on Living Conditions’  N = 2922  M:F = 58.8% male; 41.2% female  Age: 50-64 | 10 years | Original response rate 60-65%. Questionnaire data linked to registry data with passive consent. | Sex, age, marital status, education, health status, smoking, heavy alcohol intake, BMI, level of sporting activity. | Low job control was associated with increased likelihood of early retirement (HR = 1.15; C.I. 1.00 – 1.32) |
| Lund & Villadsen (2005)  NOQAG = 8 | Skill discretion (4 items), decision authority (4 items), predictability in work (2 items). | Receipt of ERP (Early Retirement Pension) during 01/01/2001 to 31/12/2014 | P: Denmark (DWECS/DREAM database)  N = 365  M:F = 54.8% male, 45.2% female  Age: 57-62 | 4 years | Not reported | Gender, age, cohabitation, social economic position, physical demands. | Low skill discretion is associated with increased early retirement (OR = 1.09; C.I. 1.00-1.19)  Predictability in work: NS |

# **Supplementary Table 7:** Evidence on work-based social support in relation to retirement intentions

| **Authors and Newcastle Ottawa Quality Assessment Grade (NOQAG)** | **Definition of psychosocial workplace characteristic** | **Retirement definition** | **Sample (population/database, N, gender balance, age range)** | **Follow up** | **Response rate** | **Adjustments** | **Direction of evidence** |
| --- | --- | --- | --- | --- | --- | --- | --- |
| Carr et al. (2016)  NOQAG = 7 | Social support (1 item) | “At what age would you like to retire?” | English Longitudinal Study of Ageing, N = 3462, male 48.8% female 51.2%, age 50-69 | 10 years | 56% of participants from wave 1 responded to all subsequent waves | Age, self-rated health, income decile, long term health problems, disability, partner’s employment status | Social support: NS |
| Hofstetter & Cohen (2014)  NOQAG = 3 | Co-worker support (10 items) | Intention to retire early (4 items) | P: Israel; unionised employees working in five well-established medium-sized industrial Israeli firms (n = 170). Surveys administered by HR representatives  N = 170  M:F = 92% male, 8% female  Age: >30 | N/A | 35% | Age, job tenure, education. | Co-worker support: NS |
| Oude Hengel et al. (2011)  NOQAG = 3 | Social support: divided into supervisor support (4 items) and co-worker support (4 items). | Willingness to work until age 65. | P: Netherlands; construction workers (Netherlands Working Conditions Surveys)  N = 5610  M:F = 100% male  Age: 30-51 | N/A | 28.1% | Age, partnership status (yes, yes with a paid job, no), shiftwork, overtime work, dangerous work, musculoskeletal symptoms. | Intermediate and low supervisor support were associated with reduced willingness to work till 65 (OR for intermediate = 0.72; C.I. 0.60-0.86; OR for low = 0.59; C.I. 0.46-0.75).  Low co-worker support was associated with increased willingness to work till 65 (OR = 1.37; C.I. 1.08-1.75). |
| Suadicani et al. (2013)  NOQAG = 3 | Social support  (6 items)  Exposure to bullying within the last year (1 item) | Intention to quit: ‘If I had the economic opportunity to do so, I would quit my job’ (yes/no) | P: Denmark; permanent staff in Bispebjerg University Hospital  N = 1809  M:F = 4% male, 96% female  Age: 45-57 (at T1) | N/A | 65% | Age, sex. | Low social support is associated with increased intention to quit job: 3 out of 6 items were significant.  Exposure to bullying within the last year was associated with increased intention to quit job (OR = 1.86; C.I. 1.28-2.69). |
| Henkens & Leenders (2010)  NOQAG = 4 | Social support from colleagues and managers | Retirement intention (4 items) | P: Netherlands, survey of four private companies and one organisation of the Dutch government  N = 2892  M:F = 76% male, 24% female  Age: 50+ | N/A | 63% | Age, sex, education, sector (public/private), tenure (full time/part time), position (executive/non-executive), replacement rate. | Social support from colleagues and managers: NS |
| Ten Have et al. (2014)  NOQAG = 6 | Low co-worker support  Low supervisor support  (both assessed using the Job Content Questionnaire) | Intention to work beyond retirement age: ‘If it was up to you, do you want to continue working after the age of 65 years?’ | P: Netherlands Mental Health Survey and Incidence Study-2  N = 1854  M:F = unclear  Age: 50-59 (at baseline) | N/A | 65.1% | Gender, age, education, partner status, having children, income situation | Low supervisor support was associated with decreased intention to work past age 65: OR = 0.60 (95% C.I. 0.42-0.85)  Low co-worker support: NS |
| Henkens & Tazelaar (1997)  NOQAG = 4 (intentions arm of the study) | Social pressure at work: "I have the feeling that my colleagues will 'force' me to retire early". | Early retirement intentions: ‘Do you intend to use the opportunity to retire early?’ (Yes/no/don't know). | P: Netherlands, postal survey of all civil servants eligible for early retirement within the following year  N = 1015  M:F = 89% male, 11% female  Age: 59 or reaching their 40^th^ year of work in 1991 | Intentions and job characteristics were recorded on the same occasion in 1991 | 79% | Financial-economic context, sex, number of dependent children, health status, normative context (whether or not the respondents partner and/or friends have already retired early), individual early-retirement valuation (would they miss particular work attributes if they retired?), perceived social support (from partner), time and information uncertainty. | Social pressure at work: NS |
| Sutinen et al. (2005)  NOQAG = 4 | Teamwork assessed using the Team Climate Inventory (14 items). | Retirement preference: ‘If it were possible to choose between work and retirement, what would you choose?’ | P: Finland; postal questionnaire sent to physicians working in three hospital districts between 1997 and 1998.  N = 447  M:F = 56.2% male, 43.8% female  Age: all ages (26-63) | N/A | 55% | Age, sex, salary, minor psychiatric morbidity. | High teamwork was associated with decreased desire to retire early (β value = -0.14). |
| Thorsen et al. (2012)  NOQAG = 4 | Quality of leadership (2 items) | Intention to retire before age 65. | P: Denmark; Danish National working Environment Study, an internet survey.  N = 3122  M:F = 47% male, 53% female  Age: >50 | N/A | 77% | Separate analyses  Gender, age,  vocational education, physical  work strain, occupational group,  socio-economic status, data collection mode, self-rated health,  depressive symptoms,  work performance  and work ability.  Combined analyses  As above, but the analysis of each variable included all covariates and the four significant psychosocial variables (ageism, lack of possibilities for development, lack of recognition, lack of predictability). | Separate analyses  Poor leadership: NS  Combined analysis of all factors  Poor leadership: NS |

# **Supplementary Table 8:** Evidence on work-based social support in relation to actual retirement

| **Authors and Newcastle Ottawa Quality Assessment Grade (NOQAG)** | **Definition of psychosocial workplace characteristic** | **Retirement definition** | **Sample (population/database, N, gender balance, age range)** | **Follow up** | **Response rate** | **Adjustments** | **Direction of evidence** |
| --- | --- | --- | --- | --- | --- | --- | --- |
| Thorsen et al. (2016)  NOQAG = 8 | Low recognition from management  Poor leadership quality  Poor social community at work  Poor trust between colleagues | Retirement age of 60-64 years as recorded in the Danish national register of social welfare beneficiaries. | DANES 2008 questionnaire survey, N = 1876, 50% male and 50% female, age 60-64 | 4 years | 66%, 69%, 77% (sample was drawn from 3 separate questionnaires) | Physical strain, socioeconomic status, gender, cohabitation, shift and night work, part-time work, sample, data collection, mental health, self-rated health | Low recognition from management was associated with increased hazard ratio for early retirement: HR = 1.87 (C.I. 1.40-2.51; p < 0.0001)  Poor leadership quality was associated with increased hazard ratio for early retirement: HR = 1.45 = (C.I. 1.04-2.04; p = 0.03)  Poor social community at work: NS  Poor trust between colleagues: NS |
| Leijten et al. (2015)  NOQAG = 5 | Support at work (4 items including both supervisor and co-worker support) | Either self-report as ‘early retirement’ or retirement before the age of 65. | Dutch longitudinal Study on Transitions in Employment, Ability and Motivation, N = 8149, male 56.6% female 43.4%, age 45-64 | 3 years | 2010 = 71% response rate (baseline)  2011 = 82% of baseline  2012 = 80% of baseline  2013 = 74 % of baseline | Age, gender, educational level | Lower support at work was associated with increased risk of early retirement: HR 1.16 (C.I. 1.00-1.35) |
| Carr et al. (2016)  NOQAG = 7 | Social support (1 item) | Actual exit from work was a shift from >0 hours of work per week to 0 hours of work per week (between two waves of data collection) | English Longitudinal Study of Ageing, N = 3462, male 48.8% female 51.2%, age 50-69 | 10 years | 56% of participants from wave 1 responded to all subsequent waves | Age, self-rated health, income decile, long term health problems, disability, partner’s employment status | Social support: NS |
| Lund et al. (2001)  NOQAG = 6 | Social support (derived from a 20 item ‘psychosocial work environment’ scale). | Self-reported receipt of early retirement pension. | P: Denmark; a sample of waste collectors and municipal workers  N = 2618  M:F = 100% male  Age: all ages | 2.5 years | 78.8% | Age, occupational group, health, smoking, marital status. | Social support: NS |
| Mein et al. (2000)  NOQAG = 6 | Social support (6 items) measured in the ‘Karasek Job Context Instrument’ | Retirement before age 59.5 years | P: UK; London based civil servants (Whitehall II Study)  N = 2532  M:F = 67% male, 33% female  Age: 50-59.5 | 7 years | 73% | Age, duration of employment in civil service, grade, job satisfaction, perceived health, marital status, material problems, housing, car access, long term illness, general health, job demands, work support | Social support  Men: high social support was associated with decreased early retirement vs low support (OR = 0.82; C.I. 0.66-1.00)  Women: NS |
| Van Solinge & Henkens (2013)  NOQAG = 3 | Perceived supervisor support for working till 65 (2 items) | Actual retirement age as established by survey. | P: Netherlands; employees of civil service and 3 large private companies  N = 1460  M:F = unclear  Age: 50-59 (at baseline) | 10 years | 62% | Partner work status, wealth, income and health, organisation. | High perceived supervisor support for working till 65 was associated with later retirement (HR = 0.838; p <0.01). |
| de Wind et al. (2015)  NOQAG = 7 | Social support from colleagues and supervisors (4 items) | Retirement before the official retirement age of 65. | P: Netherlands STREAM (Dutch Study on Transitions in Employment, Ability and Motivation)  N = 1862  M:F = 59% male; 41% female  Age: 58-62 (at baseline) | 2 years | Response rate at baseline not given; 84% at T2; 75% at T3 (of T1 sample). | Age, gender, educational level. | Higher social support was associated with increased early retirement (β = 0.14) |
| Henkens & Tazelaar (1997)  NOQAG = 7 (behaviour arm of the study) | Social pressure at work: "I have the feeling that my colleagues will 'force' me to retire early". | Actual early-retirement behaviour within 3 years of the survey. Based on administrative data (Central Salary Administration). | P: Netherlands, postal survey of all civil servants eligible for early retirement within the following year  N = 1015  M:F = 89% male, 11% female  Age: 59 or reaching their 40^th^ year of work in 1991 | 3 years | 79% | Financial-economic context, sex, number of dependent children, health status, normative context (whether or not the respondents partner and/or friends have already retired early), individual early-retirement valuation (would they miss particular work attributes if they retired?), perceived social support (from partner), time and information uncertainty. | Social pressure at work: NS |
| Lund & Villadsen (2005)  NOQAG = 8 | Social support (4 items), conflicts in work (4 items). | Receipt of ERP (Early Retirement Pension) during 01/01/2001 to 31/12/2014 | P: Denmark (DWECS/DREAM database)  N = 365  M:F = 54.8% male, 45.2% female  Age: 57-62 | 4 years | Not reported | Gender, age, cohabitation, social economic position, physical demands. | Social support: NS.  High conflict in work is associated with increased early retirement (OR = 1.43; C.I. 1.08-1.90). |
| Clausen et al. (2014)  NOQAG = 4 | Quality of leadership (4 items) | Self-reported work status: still employed/quit/retired | P: Denmark; employees from Danish Eldercare services in 35 municipalities.  N = 7025  M:F = 4% male, 96% female  Age: all ages | 18-24 months | 78% at baseline, 73.5% at follow up | None reported | Quality of leadership: NS |

# **Supplementary Table 9:** Evidence on job insecurity in relation to retirement intentions

| **Authors and Newcastle Ottawa Quality Assessment Grade (NOQAG)** | **Definition of psychosocial workplace characteristic** | **Retirement definition** | **Sample (population/database, N, gender balance, age range)** | **Follow up** | **Response rate** | **Adjustments** | **Direction of evidence** |
| --- | --- | --- | --- | --- | --- | --- | --- |
| Stynen et al. (2016)  Cross-sectional NOQAG = 5  Longitudinal NOQAG = 6 | Possibility of demotion (1 item) | “It is my intention to keep working until I reach the mandatory retirement age” | Maastricht Cohort Study (Netherlands population), N = 678, Male 90.9% female 9.1%, Age >50 (subdivided into 3 categories) | 2 years | 45% in the first year of data collection (1998). Response rate for 2012 unobtainable. | Emotional demands, gender, strenuous work, household situation, education level, economic sector | Cross sectional findings  Possibility of demotion: NS  Longitudinal findings  (NS results are not mentioned)  Age 55-59:  Possibility of demotion: (β = 0.34. S.E. = 0.19, p <0.05) |
| Ten Have et al. (2014)  NOQAG = 6 | Low job security (assessed using the Job Content Questionnaire) | Intention to work beyond retirement age: ‘If it was up to you, do you want to continue working after the age of 65 years?’ | P: Netherlands Mental Health Survey and Incidence Study-2  N = 1854  M:F = unclear  Age: 50-59 (at baseline) | N/A | 65.1% | Gender, age, education, partner status, having children, income situation | Low job security: NS |

# **Supplementary Table 10:** Evidence on job insecurity in relation to actual retirement

| **Authors and Newcastle Ottawa Quality Assessment Grade (NOQAG)** | **Definition of psychosocial workplace characteristic** | **Retirement definition** | **Sample (population/database, N, gender balance, age range)** | **Follow up** | **Response rate** | **Adjustments** | **Direction of evidence** |
| --- | --- | --- | --- | --- | --- | --- | --- |
| Lund & Villadsen (2005)  NOQAG = 8 | Job insecurity (4 items) | Receipt of ERP (Early Retirement Pension) during 01/01/2001 to 31/12/2014 | P: Denmark (DWECS/DREAM database)  N = 365  M:F = 54.8% male, 45.2% female  Age: 57-62 | 4 years | Not reported | Gender, age, cohabitation, social economic position, physical demands. | Job insecurity: NS. |

# **Supplementary Table 11:** Evidence on effort-reward imbalance in relation to retirement intentions

| **Authors and Newcastle Ottawa Quality Assessment Grade (NOQAG)** | **Definition of psychosocial workplace characteristic** | **Retirement definition** | **Sample (population/database, N, gender balance, age range)** | **Follow up** | **Response rate** | **Adjustments** | **Direction of evidence** |
| --- | --- | --- | --- | --- | --- | --- | --- |
| Siegrist et al. (2006)  NOQAG = 6 | ERI = reward (5 items) and effort (2 items), individuals were put into tertiles within their country | Intended retirement: ‘Thinking about your present job, would you like to retire as early as possible?’ [yes/no] | P: 10 European countries (SHARE dataset)  N = 6836  M:F = 51.5% male, 48.5% female  Age: from 50-65 | N/A | 55.4% (household response rate) | Country, gender, age, education, income, self-perceived health, depressive symptoms, physical symptoms, quality of life in old age. | High ERI was significantly associated with desire to retire as early as possible (OR = 1.72; C.I. 1.43-2.08) |
| Wahrendorf et al. (2013)  NOQAG = 4 | ERI = effort (2 items) divided by reward (5 items) to produce a ratio (smaller numbers = more favourable ERI). Participants in upper tertile are deemed to have high ERI. | Early retirement intentions “Thinking about your current job, would you like to retire as early as possible?” [Yes/No] | P: 11 European countries (SHARE data)  N = 6398  M:F = 55.6% male, 44.4% female  Age: 50-64 | N/A | 60.6% | Gender, age, functional limitations, country, work stress | High ERI was associated with increased early retirement intentions (OR = 1.90; p <0.001) |

# **Supplementary Table 12:** Evidence on effort-reward imbalance in relation to actual retirement

| **Authors and Newcastle Ottawa Quality Assessment Grade (NOQAG)** | **Definition of psychosocial workplace characteristic** | **Retirement definition** | **Sample (population/database, N, gender balance, age range)** | **Follow up** | **Response rate** | **Adjustments** | **Direction of evidence** |
| --- | --- | --- | --- | --- | --- | --- | --- |
| Virtanen et al. (2014)  NOQAG = 8 | Effort reward imbalance: ‘ratio between efforts spent and rewards gained’ | Remaining in employment 6 months beyond pensionable age | P: Finland; employees working in 10 towns and 21 hospitals.  N = 4677  M:F = 27.5% male, 72.5% female  Age: those who reach pensionable age between 2005-2011 | Mean = 3.7 years | 72% | Individual pensionable age, sex, socioeconomic status, marital status, residence and area, type of employment contract, work schedule, part-time pension, job strain, effort-reward imbalance, work time control, chronic disease, symptoms of ill health, smoking, alcohol use, leisure time physical activity, and obesity | Effort-reward imbalance: NS |
| Robroek et al. (2013)  NOQAG = 7 | ‘Effort–reward imbalance was defined as the country-specific upper tertile of the ratio of the sum score of the effort items and the sum of the reward items, both adjusted for the number of items’ | Early retirement = self-reported retirement before the statutory country-specific retirement age. | P: 11 European countries (SHARE dataset)  N = 4923  M:F = 56.6% male, 43.4% female  Age: >50 (average age of 55.2) | 4 years | 62% | Age, sex, education, cohabitation status, health, health behaviour, lack of physical activity, smoking, alcohol intake. | Effort-reward imbalance: NS |
| Hintsa et al. (2015)  NOQAG = 7 | Effort (6 items) and reward (11 items) scores were turned into a ratio of effort/reward | Exit from the labour market at or below age 61. | P: England; English Longitudinal Study of Aging (ELSA)  N = 1263  M:F = 52.3% male; 47.7% female  Age: 50-74 years | 6 years | Not given | Age, sex, education, occupational class, depression and allostatic load. | High ERI was associated with increased odds of early exit from labour market: OR = 1.62; C.I. 1.01- 2.61 (multiple imputed results analysis) |

# **Supplementary Table 13:** Evidence on job satisfaction in relation to retirement intentions

| **Authors and Newcastle Ottawa Quality Assessment Grade (NOQAG)** | **Definition of psychosocial workplace characteristic** | **Retirement definition** | **Sample (population/database, N, gender balance, age range)** | **Follow up** | **Response rate** | **Adjustments** | **Direction of evidence** |
| --- | --- | --- | --- | --- | --- | --- | --- |
| Burnay (2008)  NOQAG = 3 | Professional satisfaction:  not satisfied/satisfied/very satisfied | ‘Ideally, at what age would you like to retire?’ (early retirement was defined as retirement aged <60) | P: Belgium; workers from 12 different industry sectors  N = 812  M:F = 53.6% male, 46.4% female  Age: all ages | N/A | Not reported | Sex, education level, no. of children, presence of spouse, housing type, place of residence. | Low professional satisfaction was associated with increased intentions to retire early (OR = 0.55, p <0.001). |
| Pit & Hansen (2014)  NOQAG = 3 | Job satisfaction (1 item) on a scale of 0-10 | Intention to retire before age 65. | P: Australia; doctors working in general practice in the Northern Rivers region of New South Wales  N = 92  M:F = 60% male, 40% female  Age: all ages (average age of 51) | N/A | 56% | Age, sex, type of practice, years in general practice, average weekly working hours, Psychological distress, general health (SF-36). | Early retirement intentions were associated with lower levels of job satisfaction (OR = 0.74; C.I. 0.57-0.97). |
| Henkens & Tazelaar (1997)  NOQAG = 4 (intentions arm of the study) | Lack of challenge at work (2 items): 'The work I'm doing is not very challenging'; 'the work I'm doing has become more and more boring and routine'. | Early retirement intentions: ‘Do you intend to use the opportunity to retire early?’ (Yes/no/don't know). | P: Netherlands, postal survey of all civil servants eligible for early retirement within the following year  N = 1015  M:F = 89% male, 11% female  Age: 59 or reaching their 40^th^ year of work in 1991 | Intentions and job characteristics were recorded on the same occasion in 1991 | 79% | Financial-economic context, sex, number of dependent children, health status, normative context (whether or not the respondents partner and/or friends have already retired early), individual early-retirement valuation (would they miss particular work attributes if they retired?), perceived social support (from partner), time and information uncertainty. | Lack of challenge was associated with increased intentions to retire early (logit coefficient 0.41, p<0.05). |
| Hofstetter & Cohen (2014)  NOQAG = 3 | Job content plateau (6  items)  Career satisfaction (5 items) | Intention to retire early (4 items) | P: Israel; unionised employees working in five well-established medium-sized industrial Israeli firms (n = 170). Surveys administered by HR representatives  N = 170  M:F = 92% male, 8% female  Age: >30 | N/A | 35% | Age, job tenure, education | Job content plateau was associated with increased intention to retire early (r = o.31, p <0.01).  Career satisfaction: NS |
| Zappala et al. (2008)  NOQAG = 3 | Job satisfaction (satisfaction with pay, job stability, and relationships with co-workers and supervisors) | Preference for early retirement (preferred retirement age lower than expected retirement age) | P: Italy; workers from public and private sectors.  N = 218  M:F = 45% male, 55% female  Age: 45-63 | N/A | Not reported | Age, sex, annual income, health, adequacy of financial situation for retirement, negative attitude to retirement, fear of social isolation, future planning, informed on pensions. | Job satisfaction: NS |
| Damman et al. (2011)  NOQAG = 3 | Subjective work challenge (3 items) | Intention to retire early (5 items): e.g., ‘Do you intend to stop working before age 65?’ | P: Netherlands; civil servants and 3 large Dutch private sector organisation  N = 1229  M:F = 100% male  Age: >50 | N/A | 62% at baseline 75% at follow up | Organisation, age, midlife education, midlife work experiences, midlife health experiences, midlife family experiences, pre-retirement financial situation, subjective health, partner’s work status. | High subjective work challenge was associated with decreased intentions to retire early (-0.31; p <0.01). |
| Suadicani et al. (2013)  NOQAG = 3 | Job satisfaction (1 item): ‘Do you consider your job meaningful?’ | Intention to quit: ‘If I had the economic opportunity to do so, I would quit my job’ (yes/no). | P: Denmark; permanent staff in Bispebjerg University Hospital  N = 1809  M:F = 4% male, 96% female  Age: 45-57 (at T1) | N/A | 65% | Age, sex. | High/very high meaningfulness is associated with reduced desire to retire versus low meaningfulness  (OR = 0.46; C.I. 0.35-0.59). |
| Davies & Cartwright (2011)  NOQAG = 4 | Job satisfaction (16 item scale) | Willingness to work past the age of 60 (4 items) | P: UK; employees from a UK financial services organisation  N = 556  M:F = 42% male; 58% female  Age: 40-60 | N/A | 46.3% | Number of years of service, age, health, financial comfort, psychosocial expectations of retirement. | Higher satisfaction predicts a lower likelihood of intending to work beyond the age of 60 (β = -0.06; p <0.05) |
| Henkens & Leenders (2010)  NOQAG = 4 | Job challenge (3 items) | Retirement intention (4 items) | P: Netherlands, survey of four private companies and one organisation of the Dutch government  N = 2892  M:F = 76% male, 24% female  Age: 50+ | N/A | 63% | Age, sex, education, sector (public/private), tenure (full time/part time), position (executive/non-executive), replacement rate. | Higher levels of job challenge were associated with decreased intention to retire early (β = -0.15, p <0.001). |
| Kilty & Behling (1985)  NOQAG = 5 | ‘Work provides active interest’ (5 items)  ‘Work gives meaning to life’ (3 items)  ‘Satisfaction with career’ (6 items) | Age at which participants plan to retire (1 item)  Whether participants have considered early retirement (1 item) | P: America; a sample of lawyers, social workers, college professors and high school teachers from Ohio  N = 457  M:F = 52% male, 48% female  Age: 25-64 | N/A | Not reported | Age, sex, profession (attorney, social worker, high school teacher), years of education, marital status, have a dependent child, self-employed, number of jobs, timing current position, household income, hours working. | Considered early retirement?  ‘Work provides active interest’ was associated with decreased consideration of early retirement (β = -0.17; p <0.05)  ‘Work gives meaning to life’ was associated with decreased consideration of early retirement (β = -0.09; p <0.05)  Other variables were not significant for this outcome.  Age of planned retirement  ‘Work provides active interest’ was associated with later age of intended retirement (β = 0.19; p <0.05)  Higher satisfaction with career was associated with earlier age of intended retirement (β = -0.16; p <0.05)  ‘Work gives meaning to life’ was associated with later age of intended retirement (β = 0.14; p <0.05) |
| Oakman & Wells (2012)  NOQAG = 3 | Job satisfaction (15 items) | What is your intended timing of retirement? (less than 5 years or more than 5 years). | P: Australia; questionnaires distributed via the intranet in a large public services company in Victoria.  N = 332  M:F = 61% male, 39% female  Age: All ages (average 43.5) | N/A | 47.4% | Age, gender, length of service, marital status, dependent children. | High job satisfaction was correlated with decreased intention to retire in the next 5 years (OR = 0.67; 0.46-0.97). |
| Schreurs et al. (2011a)  NOQAG = 4 | Work enjoyment (5 items). | Early retirement intention (4 items). | P: Belgium; stratified sample from large online panel of Belgian active working population  N = 1812  M:F = 62% male, 38% female  Age: >45 (average age of 48) | N/A | 38.8% | Sex, education, occupational status (stratified for age). | High work enjoyment was associated with decreased intention to retire early (γ = -0.32, p <0.01) |
| Van den Berg (2011)  NOQAG = 3 | Work challenge (3 items) | Willingness to continue working (2 items) | P: Netherlands; participants recruited while attending training classes at a professional knowledge centre.  N = 73  M:F = 52% male, 48% female  Age: 50-65 | N/A | 45% | Sex, age, job tenure | High work challenge was significantly associated with willingness to continue working (β = 0.42; C.I. 0.24-0.60, p < 0.05) |

# **Supplementary Table 14:** Evidence on job satisfaction in relation to actual retirement

| **Authors and Newcastle Ottawa Quality Assessment Grade (NOQAG)** | **Definition of psychosocial workplace characteristic** | **Retirement definition** | **Sample (population/database, N, gender balance, age range)** | **Follow up** | **Response rate** | **Adjustments** | **Direction of evidence** |
| --- | --- | --- | --- | --- | --- | --- | --- |
| Thorsen et al. (2016)  NOQAG = 8 | Low job satisfaction | Retirement age of 60-64 years as recorded in the Danish national register of social welfare beneficiaries. | DANES 2008 questionnaire survey, N = 1876, 50% male and 50% female, age 60-64 | 4 years | 66%, 69%, 77% (sample was drawn from 3 separate questionnaires) | Physical strain, socioeconomic status, gender, cohabitation, shift and night work, part-time work, sample, data collection, mental health, self-rated health | Low job satisfaction was associated with increased hazard ratio of early retirement: HR = 3.33 (C.I. 2.36-4.70; p < 0.0001) |
| Henkens & Tazelaar (1997)  NOQAG = 7 (behaviour arm of the study) | Lack of challenge at work (2 items): 'The work I'm doing is not very challenging'; 'the work I'm doing has become more and more boring and routine'. | Actual early-retirement behaviour within 3 years of the survey. Based on administrative data (Central Salary Administration). | P: Netherlands, postal survey of all civil servants eligible for early retirement within the following year  N = 1015  M:F = 89% male, 11% female  Age: 59 or reaching their 40^th^ year of work in 1991 | 3 years | 79% | Financial-economic context, sex, number of dependent children, health status, normative context (whether or not the respondents partner and/or friends have already retired early), individual early-retirement valuation (would they miss particular work attributes if they retired?), perceived social support (from partner), time and information uncertainty. | Lack of challenge was associated with increased early retirement behaviour (logit coefficient 0.32, p<0.05). |
| Kubicek et al. (2010)  NOQAG = 6 | Job satisfaction: ‘all things considered, how satisfied are you with your job as a whole?’ | Self-reported actual retirement (telephone interview). Early retirement is retirement at or before age 62. | P: USA (Wisconsin Longitudinal Study); a random sample of graduates of Wisconsin high schools in 1957, participants had to be married at baseline and follow up, and they had to have worked for at least 10 years  N = 2499  M:F = 55.7% male, 44.3% female  Age: 51-56 at baseline | 11 years | Not reported | Pension plan, health insurance, spouses pension plan, spouse’s health insurance, educational attainment, income, occupational education, marital satisfaction (a factor comprised of family-to-work conflict, family resources, and family demands). | High job satisfaction was associated with decreased early retirement (β = -0.07, p <0.05). |
| Damman et al. (2011)  NOQAG = 5 | Subjective work challenge (3 items) | Retirement before age 65 | P: Netherlands; civil servants and 3 large Dutch private sector organisation  N = 1229  M:F = 100% male  Age: >50 | 6 years | 62% at baseline 75% at follow up | Organisation, age, midlife education, midlife work experiences, midlife health experiences, midlife family experiences, pre-retirement financial situation, subjective health, partner’s work status | High subjective work challenge was associated with decreased early retirement (-0.34; p <0.01). |
| Mein et al. (2000)  NOQAG = 6 | Job satisfaction: ‘About your job in general. How satisfied are you with your job as a whole, taking everything into consideration?’ | Retirement before age 59.5 years | P: UK; London based civil servants (Whitehall II Study)  N = 2532  M:F = 67% male, 33% female  Age: 50-59.5 | 7 years | 73% | Age, duration of employment in civil service, grade, job satisfaction, perceived health, marital status, material problems, housing tenure, car access, long-term illness, general health questionnaire score, job demands, work support. | Men: lack of job satisfaction was associated with increased early retirement (OR = 1.99; C.I. 1.56-2.53)  Women: lack of job satisfaction was associated with increased early retirement (OR = 1.63; C.I. 1.12-2.38) |
| Van Solinge & Henkens (2013)  NOQAG = 3 | Job challenge (3 items) | Actual retirement age as established by survey. | P: Netherlands; employees of civil service and 3 large private companies  N = 1460  M:F = unclear  Age: 50-59 (at baseline) | 10 years | 62% | Partner work status, wealth, income and health, organisation. | Job challenge: NS |
| Lund & Villadsen (2005)  NOQAG = 8 | Reward in work (3 items), meaning of work (3 items). | Receipt of ERP (Early Retirement Pension) during 01/01/2001 to 31/12/2014 | P: Denmark (DWECS/DREAM database)  N = 365  M:F = 54.8% male, 45.2% female  Age: 57-62 | 4 years | Not reported | Gender, age, cohabitation, social economic position, physical demands. | Both variables were not significant. |
| Clausen et al. (2014)  NOQAG = 4 | Experience of meaning at work (3 items) | Self-reported work status: still employed/quit/retired | P: Denmark; employees from Danish Eldercare services in 35 municipalities.  N = 7025  M:F = 4% male, 96% female  Age: all ages | 18-24 months | 78% at baseline, 73.5% at follow up | None | Experience of meaning at work: NS |
| Robroek et al. (2013)  NOQAG = 7 | Job rewards (5 items) | Early retirement = self-reported retirement before the statutory country-specific retirement age. | P: 11 European countries (SHARE dataset)  N = 4923  M:F = 56.6% male, 43.4% female  Age: >50 (average age of 55.2) | 4 years | 62% (household response rate) | Age, sex, education, cohabitation status, health, health behaviour, lack of physical activity, smoking, alcohol intake. | Job rewards: NS |

# **Supplementary Table 15:** Evidence on organizational resources in relation to retirement intentions

| **Authors and Newcastle Ottawa Quality Assessment Grade (NOQAG)** | **Definition of psychosocial workplace characteristic** | **Retirement definition** | **Sample (population/database, N, gender balance, age range)** | **Follow up** | **Response rate** | **Adjustments** | **Direction of evidence** |
| --- | --- | --- | --- | --- | --- | --- | --- |
| Hofstetter & Cohen (2014)  NOQAG = 3 | Perceived organisational support (12 items), short version of the Survey of Perceived Organisational Support. | Intention to retire early (4 items) | P: Israel; unionised employees working in five well-established medium-sized industrial Israeli firms (n = 170). Surveys administered by HR representatives  N = 170  M:F = 92% male, 8% female  Age: >30 | N/A | 35% | Age, job tenure, education. | Perceived organisational support was associated with decreased early retirement intentions (r = -0.33, p ,0.01). |
| Sutinen et al. (2005)  NOQAG = 4 | Organisational justice (13 items). | Retirement preference: ‘If it were possible to choose between work and retirement, what would you choose?’ | P: Finland; postal questionnaire sent to physicians working in three hospital districts between 1997 and 1998.  N = 447  M:F = 56.2% male, 43.8% female  Age: all ages (26-63) | N/A | 55% | Age, sex, salary, minor psychiatric morbidity. | Organisational justice: NS |
| Van den Berg (2011)  NOQAG = 3 | Organisational stimulation (4 items) | Willingness to continue working (2 items) | P: Netherlands; participants recruited while attending training classes at a professional knowledge centre.  N = 73  M:F = 52% male, 48% female  Age: 50-65 | N/A | 45% | Sex, age, job tenure | High organisational stimulation was significantly associated with willingness to continue working (β = 0.40; C.I. 0.22-0.58, p < 0.05) |
| Heponiemi et al. (2008)  NOQAG = 3 | Organisational injustice, a scale comprised of four dimensions: procedural injustice (7 items), interpersonal justice (4 items), informational justice (5 items), and distributed justice (4 items). | Strength of retirement intentions (3 levels) based on two items | P: Finland, physicians drawn from the database of the Finnish Medical Association  N = 1383  M:F = 51% male, 49% female  Age: 45-65 | N/A | 57% | Age, sex, employment sector (health centre, hospital, other), self-rated health, sickness absence within 12 months. | High organisational injustice was associated with increased intentions to retire early (OR = 1.27; 95% C.I.1.17-1.39) |

# **Supplementary Table 16:** Evidence on organizational resources in relation to actual retirement

| **Authors and Newcastle Ottawa Quality Assessment Grade (NOQAG)** | **Definition of psychosocial workplace characteristic** | **Retirement definition** | **Sample (population/database, N, gender balance, age range)** | **Follow up** | **Response rate** | **Adjustments** | **Direction of evidence** |
| --- | --- | --- | --- | --- | --- | --- | --- |
| Thorsen et al. (2016)  NOQAG = 8 | Poor trust in management  Low organisational justice | Retirement age of 60-64 years as recorded in the Danish national register of social welfare beneficiaries. | DANES 2008 questionnaire survey, N = 1876, 50% male and 50% female, age 60-64 | 4 years | 66%, 69%, 77% (sample was drawn from 3 separate questionnaires) | Physical strain, socioeconomic status, gender, cohabitation, shift and night work, part-time work, sample, data collection, mental health, self-rated health | Poor trust in management was associated with increased hazard ratio for early retirement: HR = 1.45 (C.I. 1.04-2.03; p = 0.03)  Low organisational justice was associated with an increased hazard ratio for early retirement: HR = 1.55 (C.I. 1.05-2.31; p = 0.03) |
| Lund & Villadsen (2005)  NOQAG = 8 | Management quality (4 items) | Receipt of ERP (Early Retirement Pension) during 01/01/2001 to 31/12/2014 | P: Denmark (DWECS/DREAM database)  N = 365  M:F = 54.8% male, 45.2% female  Age: 57-62 | 4 years | Not reported | Gender, age, cohabitation, social economic position, physical demands. | Management quality: NS |
